# Mechanical response and acoustic emission characteristics of damaged mudstone

**DOI:** 10.1038/s41598-025-23550-6

**Published:** 2025-11-13

**Authors:** Peng Di

**Affiliations:** 1 China Nonferrous Metals (Guilin) Geology and Mining Co., Ltd., Guilin, 541004 Guangxi China; 2 China Coal Geology Group Co., Ltd., Beijing, 100040 China

**Keywords:** Damaged mudstone, Mechanical response, Strain energy, Acoustic emission, Crack propagation, Engineering, Materials science, Natural hazards

## Abstract

Mudstone, which is a representative weak rock mass, often experiences mechanical deterioration because of repeated stress disturbances in underground engineering. This study elucidates the mechanical and acoustic emission (AE) characteristics of damaged mudstone. Cylindrical mudstone samples with damage levels corresponding to 0%, 20%, 40%, and 60% uniaxial compressive strength (UCS) were prepared and tested under uniaxial compression with real-time AE monitoring. The results reveal that the peak strength decreased with increasing damage level, whereas the elastic modulus decreased from 4.861 to 3.871 GPa. An accelerated reduction in both the peak strength and elastic modulus occurred at damage levels of 40% and 60% UCS, corresponding to a transition from slow microcrack initiation to localized crack coalescence. Energy analysis revealed that the energy inflection point appeared earlier with increasing predamage level. Both the total input energy and elastic strain energy before the peak decreased significantly, whereas the proportion of dissipated energy increased from 13.45 to 55.42%. The highly damaged mudstone exhibited step-like surges, indicating cascading crack-cluster propagation and shear‒slip localization. In terms of the AE behavior, higher predamage levels resulted in earlier activation of the AE counts and more distinct multistage bursts before the peak. AE analysis indicated that while tensile failure was dominant, the proportion of shear failure increased from 21.36 to 35.10% with increasing damage level, as indicated by a decrease in the wave velocity and a shift in the AE parameters. Moreover, the increased microcrack density and bedding plane weakness resulted in an increase in the shear failure percentage. Furthermore, the AE b value decreased from 1.089 to 0.680, and the AE S value increased from 0.202 to 0.281, confirming a shift from distributed small-scale cracking to a concentrated, large-scale fracture. These findings provide crucial quantitative insights into the mechanical degradation of damaged mudstone and valuable AE-based precursors for failure, which have important implications for hazard prediction in engineering practices involving mudstone.

## Introduction

Mudstone is extensively distributed in coal mine roadways, metal mine tunnels, underground energy storage projects, and slope^1–5^. As an important component of weak surrounding rock, its mechanical stability is vital to engineering safety^6–10^. In practical engineering, mudstone surrounding rock is frequently subjected to complex stress disturbances, such as mining-induced stress fluctuations, seismic waves, and construction activities, and thus inevitably experiences multiple loading and unloading cycles^11–15^. These stress-induced cycles not only degrade the load-bearing capacity of mudstone but also promote the initiation and propagation of cracks within it, thereby significantly reducing its structural stability. Therefore, an in-depth understanding of the mechanical response and damage evolution of damaged mudstone is of great theoretical and engineering significance for hazard prediction and support design.

In recent years, extensive studies have been conducted on the mechanical properties of various rock types under loading conditions, including sandstone, granite, and shale^16–20^. Zhu et al.^21^, by conducting constant-amplitude cyclic loading and acoustic emission (AE) tests, systematically analyzed the mechanical properties, failure modes, and AE responses of red and green sandstone under reservoir water level fluctuations in both dry and saturated conditions. Their results indicated that water saturation significantly reduces peak strength, increases failure strain, and raises the proportion of tensile failure. Zhang et al.^22^ carried out true triaxial compression and cyclic loading and unloading tests on sandstone under different loading paths, analyzing the variations in peak principal stress, fragmentation characteristics, fractal dimension, and energy evolution. The results demonstrated that graded cyclic loading causes the most severe damage to rock. Ye et al.^23^ elucidated the creep deformation and damage evolution behavior of shale, finding that the accelerated creep stage corresponds to the crack damage stress level, and developed a novel nonlinear viscoelastic–plastic direct shear creep model that incorporates time-scale effects and cohesion evolution. Chen et al.^24^ analyzed rock failure precursors from the perspectives of energy, AE, and wave velocity, concluding that high-amplitude, high-count AE events, a continuous increase in stress accompanied by a drop in wave velocity, and the linear relationship between wave velocity reduction and dissipated energy are effective precursors. Wang et al.^25^ revealed that silty mudstone exhibits a significant strengthening effect under cyclic loading between the crack initiation stress and the damage stress; however, exceeding the damage stress leads to fatigue failure, with a fatigue strength of approximately 80%–89% of the uniaxial compressive strength, and the fatigue life shows a logarithmic negative linear relationship with the upper load limit. Fu et al.^26^, through stress-controlled uniaxial cyclic loading tests on Leiyang marble, found that cyclic loading can both compact pre-existing cracks and generate new cracks, resulting in rock strengthening and stiffening, and leading to a higher crack density and energy density prior to failure, making it more prone to fragmentation than under monotonic loading.

These studies generally indicate that cyclic loading leads to reductions in peak strength and elastic modulus, accumulation of residual deformation, increased energy dissipation, and accelerated crack propagation. However, compared with hard and brittle rocks, mudstone (a fine-grained sedimentary rock) possesses distinct characteristics such as pronounced bedding structures, high clay mineral content, and low strength, making its damage evolution mechanisms more susceptible to factors including moisture, stress history, and initial defects. For example, Li et al.^27^ systematically analyzed the microstructural degradation, mechanical property deterioration, and crack propagation characteristics of carbonaceous mudstone under wet–dry cycling. Zheng et al.^28^ conducted cyclic triaxial loading tests on non-penetrative jointed mudstone with dip angles of 15°, 30°, and 45° at different frequencies. The results showed that higher frequencies yielded greater average loading and unloading elastic moduli and larger maximum axial strain differences; in contrast to triaxial compression, cyclic loading generated more cracks, with crack propagation extending from the joint tip toward both ends of the specimen. Under low-frequency conditions, additional crack development occurred after failure. Li et al.^29^, through uniaxial compression and graded-loading creep tests at various loading rates, revealed the deformation, strength, and creep behaviors of mudstone under changes in loading rate, highlighting a marked isokinetic viscous effect. Overall, previous research has largely focused on the mechanical behavior of mudstone under single loading scenarios or natural conditions, whereas systematic studies on the subsequent mechanical deterioration and failure mechanisms of mudstone under initial damage induced by preloading and unloading remain relatively limited^30^.

Compared to conventional techniques such as stress–strain monitoring, strain gauges, and wave velocity measurement, AE monitoring offers several distinct advantages in analyzing rock failure processes^31–33^. Whereas strain gauges and displacement sensors detect deformation only after a significant structural response has occurred, AE monitoring captures the initiation and dynamic propagation of microcracks in real time, often long before macroscopic failure occurs^34,35^. As a highly sensitive, non-destructive testing method, AE technology enables real-time tracking of microcrack initiation, growth, and coalescence, and has been extensively employed in studies on rock damage evolution and failure precursors^36–38^. Numerous investigations have demonstrated that variations in AE parameters can reflect crack types and evolution stages, making AE a critical tool for identifying the critical instability state^39,40^. However, most existing AE studies on mudstone have been limited to intact specimens or monotonic loading conditions^41,42^. Comprehensive analyses addressing the AE response characteristics, crack evolution patterns, and failure precursors of mudstone with different initial damage levels under subsequent cyclic loading are still lacking. In particular, further research is required to elucidate how the integration of mechanical responses and AE multi-parameter characteristics can reveal the mechanisms by which initial damage affects failure mode transitions and energy evolution in mudstone.

However, most existing studies have primarily focused on the behavior of intact rocks. Systematic investigations on the mechanical degradation laws, energy evolution patterns, and multi-parameter AE responses of mudstone with controlled initial damage levels are still limited. This study presents a novel and comprehensive analysis of mudstone subjected to controlled single preloading and unloading cycles to simulate initial damage, followed by uniaxial compression with real-time AE monitoring. In contrast to previous research, this work integrates crack classification, energy partitioning, and a wave velocity-based damage index to establish a multi-source diagnostic framework for characterizing deterioration mechanisms. These innovations bridge the gap between microstructural degradation under damage and AE-based failure prediction, thereby enhancing the applicability of findings to early warning strategies and support system design in weak rock engineering.

In this study, mudstone specimens with different initial damage levels were prepared by controlling the degree of single loading and unloading, followed by uniaxial compression tests combined with AE monitoring. The stress–strain behavior, strength degradation, energy distribution patterns, and AE evolution characteristics of mudstone with varying initial damage were systematically analyzed. The influence of initial damage on mechanical deterioration and deformation accumulation was revealed, and the evolution of AE multi-parameter characteristics and the distribution of crack types under different damage conditions were examined. The findings offer valuable theoretical insights for evaluating the stability of deep weak surrounding rock, improving disaster prediction, and optimizing support design.

## Methods

### Experimental specimens

All experimental specimens were obtained from a single block of intact mudstone. Sampling locations were carefully selected to avoid structural weak zones such as joints, faults, and weathered areas, thereby minimizing variability due to macroscopic defects. Rock cores with a diameter of Φ50 mm were drilled from intact surrounding rock and processed into cylindrical specimens (50 mm × 100 mm) in accordance with ISRM standards. The end-face unevenness was controlled within 0.02 mm, and the perpendicular deviation between the end face and the axis did not exceed 0.001 rad. To prevent moisture loss, specimens were stored at 20 °C and sealed with double layers of plastic wrap and aluminum foil. Preliminary testing showed that the average uniaxial compressive strength (UCS) of the original intact mudstone was approximately 18 MPa.

After preparing the intact specimens, different damage levels were introduced as required. Specifically, intact specimens were placed in a uniaxial testing machine and subjected to a single loading and unloading process to generate microcracks within the specimens, thereby producing varying damage levels. This was accomplished by loading the specimen to a predetermined proportion of its peak strength and then unloading, thereby generating irreversible microcracks corresponding to specific damage levels^43,44^. By varying the loading peak proportion (20%, 40%, and 60% UCS), mudstone specimens of different damage grades were prepared for subsequent tests^45^.

To accurately characterize the internal structural state of specimens at varying damage levels, ultrasonic testing was conducted to measure the longitudinal wave velocity of each mudstone specimen after predamage treatment. The results are presented in Table 1. Overall, with increasing predamage loading proportion, the reduction in wave velocity progressively increased, indicating a gradual rise in microcrack density within the specimens. Consequently, the degree of damage intensified, and the wave velocity measurements served as an important reference index for damage classification^46^.

1 $$D_{v} = 1 - \left( {\frac{{v_{1} }}{{v_{0} }}} \right)^{2}$$

where* v*_0_ denotes the longitudinal wave velocity before damage, *v*_1_ represents the longitudinal wave velocity after damage, and *D*_*v*_ indicates the damage degree of the mudstone.

**Table 1:** Basic parameters of samples.

Group	No	Mass (g)	*v*_0_ (km/s)	*v*_1_ (km/s)	Preloading degree	*D*_*v*_
Group I	#1	417.34	3.54	3.54	0% UCS	0
	#2	418.61	3.55	3.23	20% UCS	0.172
	#3	417.15	3.53	3.04	40% UCS	0.258
	#4	417.37	3.54	2.76	60% UCS	0.392
Group II	#5	416.83	3.52	3.52	0% UCS	0
	#6	417.35	3.54	3.21	20% UCS	0.178
	#7	417.81	3.55	2.99	40% UCS	0.291
	#8	416.97	3.52	2.69	60% UCS	0.416
Group III	#9	418.12	3.55	3.55	0% UCS	0
	#10	417.26	3.54	3.18	20% UCS	0.193
	#11	417.52	3.54	3.01	40% UCS	0.277
	#12	417.73	3.54	2.67	60% UCS	0.431

Furthermore, to elucidate the influence of initial damage on the subsequent mechanical behavior of mudstone, a control group with no initial damage (i.e., 0% UCS damage level) was included. These undamaged specimens were not subjected to any preloading and were directly incorporated into the subsequent experimental program. The basic information and damage levels of the mudstone specimens used in this study are provided in Table 1. Three specimens were tested for each predamage level (0%, 20%, 40%, 60% UCS) to ensure the reproducibility of the observed trends.

### Experimental equipment and procedure

**Fig. 1:**
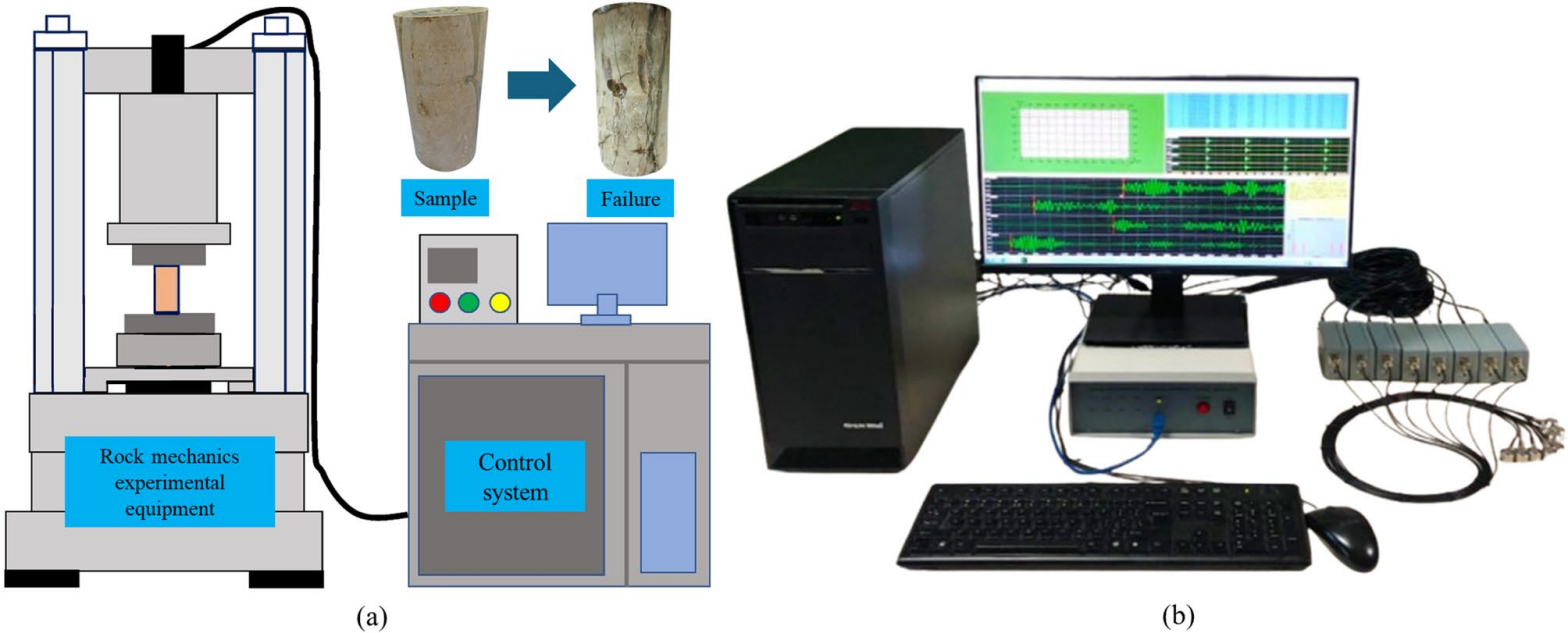
Experimental equipment. (**a**) Rock mechanics testing apparatus^47–49^; (**b**) DS-2 AE system.

As shown in Fig. 1a, to comprehensively evaluate the mechanical response and AE characteristics of damaged mudstone, mechanical experiments were conducted using a “microcomputer-controlled electronic universal testing machine” (Shenzhen Sansi Zongheng). This equipment offers control of both force and displacement and supports multiple loading modes, including constant-stress, constant-displacement, and constant-strain control. During testing, the system continuously acquired and recorded key parameters such as axial stress and axial strain throughout the loading process, providing fundamental data for subsequent mechanical behavior analysis.

AE signal acquisition and analysis were conducted using a DS-2 AE monitoring system equipped with resonant-type sensors and a multi-channel high-speed data acquisition module (Fig. 1b). The sampling frequency was set to 3 MHz, and the threshold for event triggering was 40 dB to eliminate environmental noise. Each AE sensor was connected to a preamplifier with a gain of 40 dB, and signals were transmitted via shielded low-noise coaxial cables to the acquisition unit. Two AE sensors were symmetrically mounted on the lateral surface of each cylindrical specimen, at mid-height, using a high-viscosity silicone grease as the coupling medium to ensure efficient signal transmission. The sensors were held in place with rubber bands. The AE system and the loading system were time-synchronized, ensuring accurate temporal correspondence between mechanical data (stress–strain) and AE signals.

To investigate the mechanical response and AE characteristics of damaged mudstone under uniaxial compression, displacement-controlled loading was applied at a constant rate of 0.02 mm/min. During specimen installation, each cylindrical mudstone sample was positioned between the upper and lower platens of the electronic universal testing machine with careful alignment of the loading and specimen axes. A leveling device was used to eliminate initial eccentric stress. AE monitoring was conducted using the DS-2 system, with AE sensors symmetrically arranged along the specimen height. Silicone grease was applied as a coupling agent to ensure high-quality signal transmission, and the sensors were connected to the preamplifier via shielded cables. Throughout the loading process, the mechanical testing system continuously recorded axial load and axial/radial strain, while the AE system simultaneously acquired parameters such as event amplitude, rise time, duration, ringing counts, and energy. All data were synchronously stored with time as the reference, enabling subsequent time-domain correlation analysis between the stress–strain curve and AE parameter evolution. The low loading rate effectively suppressed inertial effects, allowing for clear observation of crack initiation and propagation, and facilitating the capture of detailed temporal information on damage evolution.

## Results and analysis

### Strength degradation characteristics

**Fig. 2:**
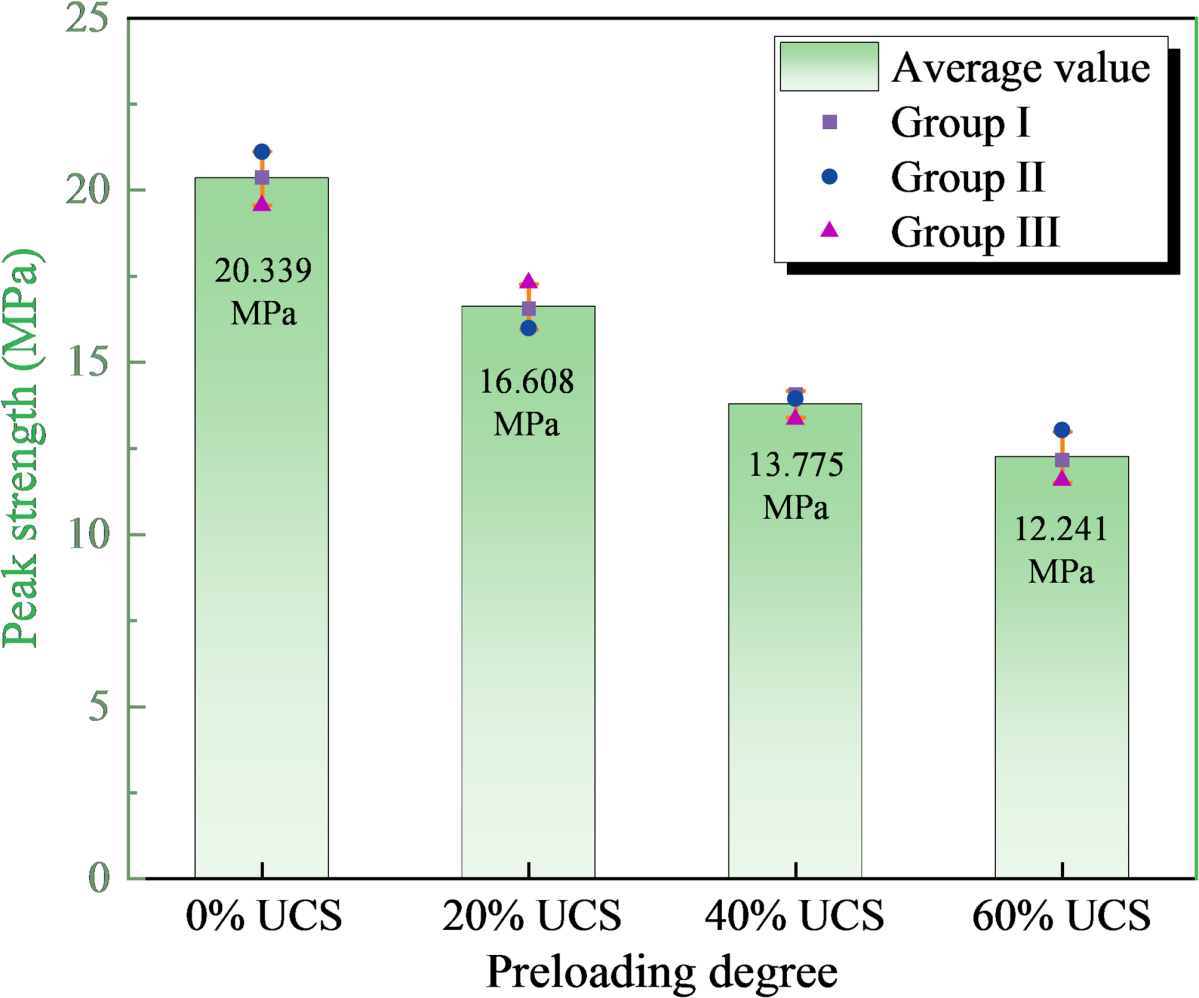
Strength degradation characteristics of damaged mudstone.

Figure 2 demonstrates the comparison of the peak strength of each group of mudstone specimens with different damage levels, with the mean values shown as a bar graph and the data for each group presented as a scatter plot. The strength degradation characteristics of mudstone with increasing damage levels can be obtained from Fig. 2. As the damage level increased, the peak strength of mudstone specimens gradually decreased, and the average values were 20.339 MPa, 16.608 MPa, 13.775 MPa and 12.241 MPa, respectively, which indicated that a varying number of microcracks were formed inside the mudstone after experiencing different damage levels. With the accumulation and connection of these microcracks, the load-bearing skeleton of the specimen was weakened, and the overall strength thus showed a decreasing trend step by step.

The results of each group of experimental data for the strength characterization of the damaged mudstone specimens are similar. Therefore, considering the limited length of the article, the subsequent experimental data analyses are only for the damaged mudstone specimens #1, #2, #3, and #4.

**Fig. 3:**
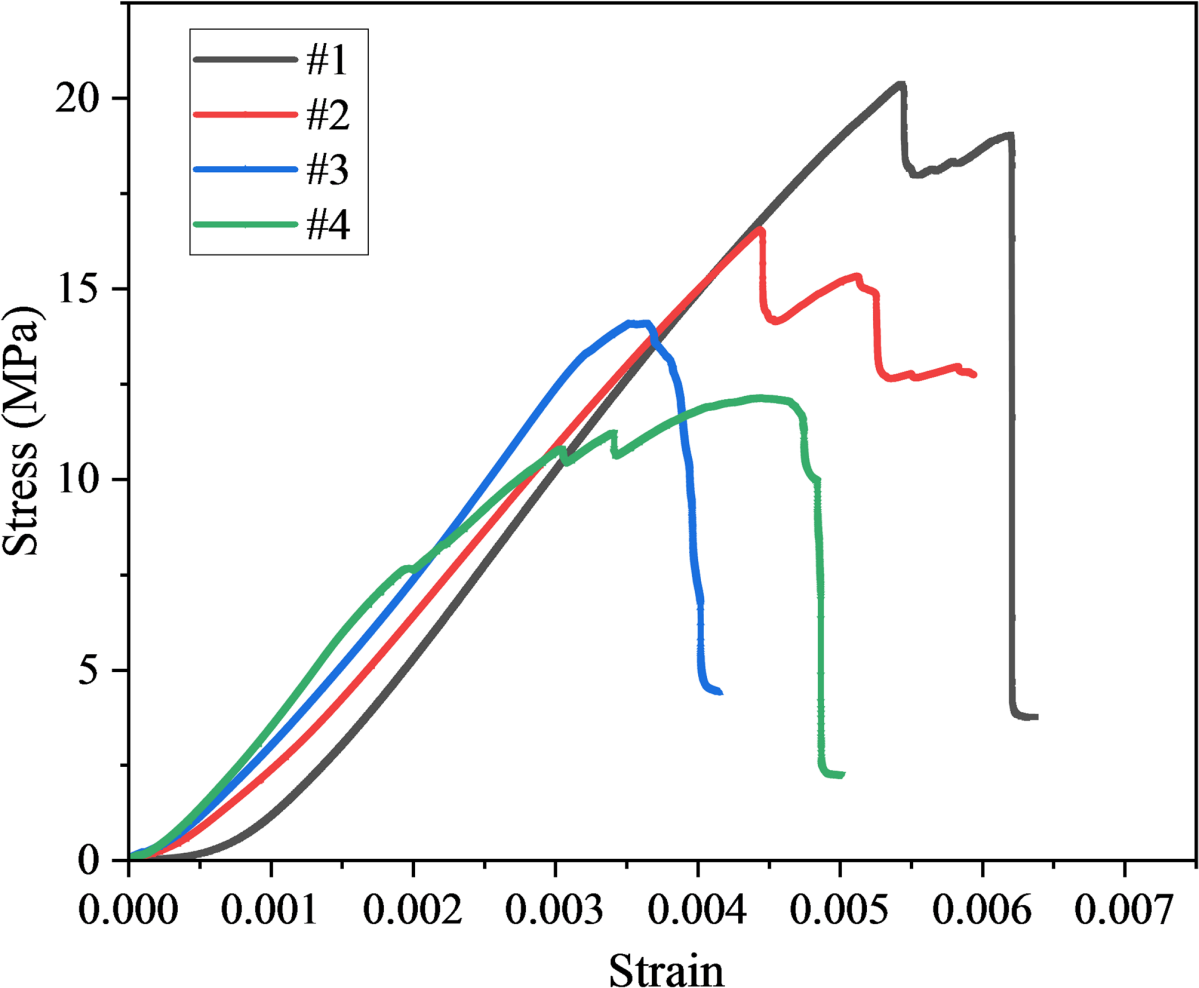
Stress–strain curves of damaged mudstone.

Figure 3 shows the stress–strain curves of undamaged and damaged mudstone specimens under uniaxial compression. Overall, all curves display the characteristic deformation and failure stages of rock in uniaxial compression, including the initial compaction stage, the near-linear elastic stage, the nonlinear yield stage, and the post-peak failure stage. With increasing initial damage levels, however, notable changes are observed in curve morphology, peak strength, and ductility, clearly reflecting the progressive deterioration of mudstone mechanical properties during damage evolution.

In the initial compaction stage, all groups show a slow stress increase with a low slope, mainly corresponding to the closure of primary pores and microcracks in the specimen. For the high-damage group (60% UCS), the duration of the compaction stage is longer, although the slope of this segment appears slightly higher than in lower damage groups. This is likely due to the presence of stiffer unbroken fragments resisting closure in the early loading phase, despite greater overall internal cracking. In the elastic stage, the stress–strain curves exhibit an approximately linear trend, with the slope corresponding to the elastic modulus. The undamaged group exhibits the highest elastic modulus, indicating good microstructural integrity and strong load-bearing capacity. As the damage level increases, the elastic modulus decreases progressively, with the high-damage group showing the most pronounced reduction, reflecting a weakening of overall stiffness. This degradation in stiffness is closely associated with the initiation and propagation of microcracks and the consequent weakening of intergranular bonding strength induced by preloading.

In the yield and peak stages, the undamaged mudstone shows the highest peak strength and peak strain, with peak strength far exceeding that of the medium- and high-damage groups. Its curve shows little fluctuation prior to the peak, indicating that crack initiation predominantly occurs within the short interval immediately preceding failure. By contrast, the medium-damage group (40% UCS) and the high-damage group (60% UCS) exhibit fluctuations before the peak, suggesting that internal cracks become active and propagate unstably at lower stress levels, leading to an earlier reduction in load-bearing capacity. The low-damage group (20% UCS) reaches a peak strength intermediate between that of the undamaged and medium-damage groups, indicating that light damage has only a limited effect on peak strength, though it still promotes earlier activation of partial microcrack activity.

The post-peak behavior reveals a clear contrast among the specimens. In the undamaged group, stress declines abruptly after reaching the peak, dropping almost instantaneously to zero, which is characteristic of a brittle failure mode. In the damaged groups, post-peak stress decreases more gradually and exhibits multiple step-like drops, indicating that failure is accompanied by uneven crack propagation and residual load release, displaying a certain degree of plasticity.

**Fig. 4:**
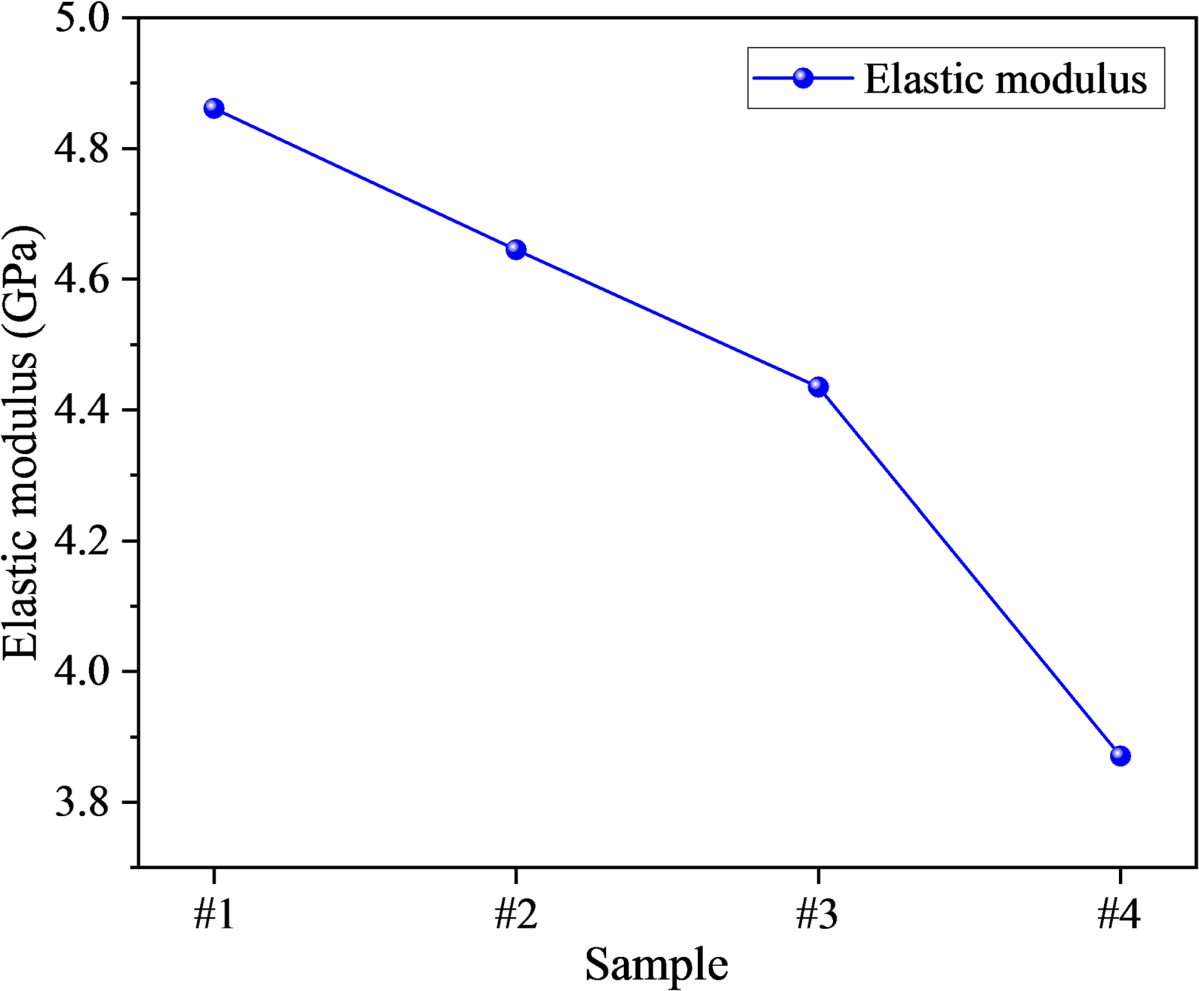
Variation of elastic modulus of damaged mudstone.

Moreover, the elastic modulus for each specimen was obtained by linearly fitting the stress–strain curve between 30 and 70% of the peak stress, where the response was approximately linear. This method eliminates the compaction and yield stages, providing a consistent basis for comparison^50,51^. As shown in Fig. 4, the elastic modulus of the damaged mudstone specimens were 4.861 GPa, 4.645 GPa, 4.435 GPa, and 3.871 GPa, respectively. The elastic modulus also decreased with increasing damage levels and exhibited a segmented degradation pattern: it declined approximately linearly in the 0–40% damage range, whereas the degradation rate increased significantly in the 40–60% range. This reflects a transition in damage mode from slow microcrack initiation to localized crack coalescence. The extracted values were 4.861 GPa for specimen #1 and 3.871 GPa for specimen #4. Although the modulus difference appears visually subtle in Fig. 2 due to scale compression and overlapping slopes, the regression analysis confirms a measurable degradation in stiffness.

The observed deterioration is primarily attributed to the formation of numerous microcracks during the preloading process, which increased porosity and crack density, reduced the effective load-bearing area, and induced local stress concentrations that facilitated earlier crack initiation and accelerated propagation at lower stress levels. Moreover, owing to the well-developed bedding structure of mudstone, the contact stiffness among grains, matrix, and bedding planes underwent irreversible degradation during loading, while interfacial friction and bonding strength decreased. At higher damage levels, micro-shear slip along bedding planes evolved into through-going slip surfaces, thereby accelerating the reduction of both modulus and strength.

### Energy evolution characteristics

**Fig. 5:**
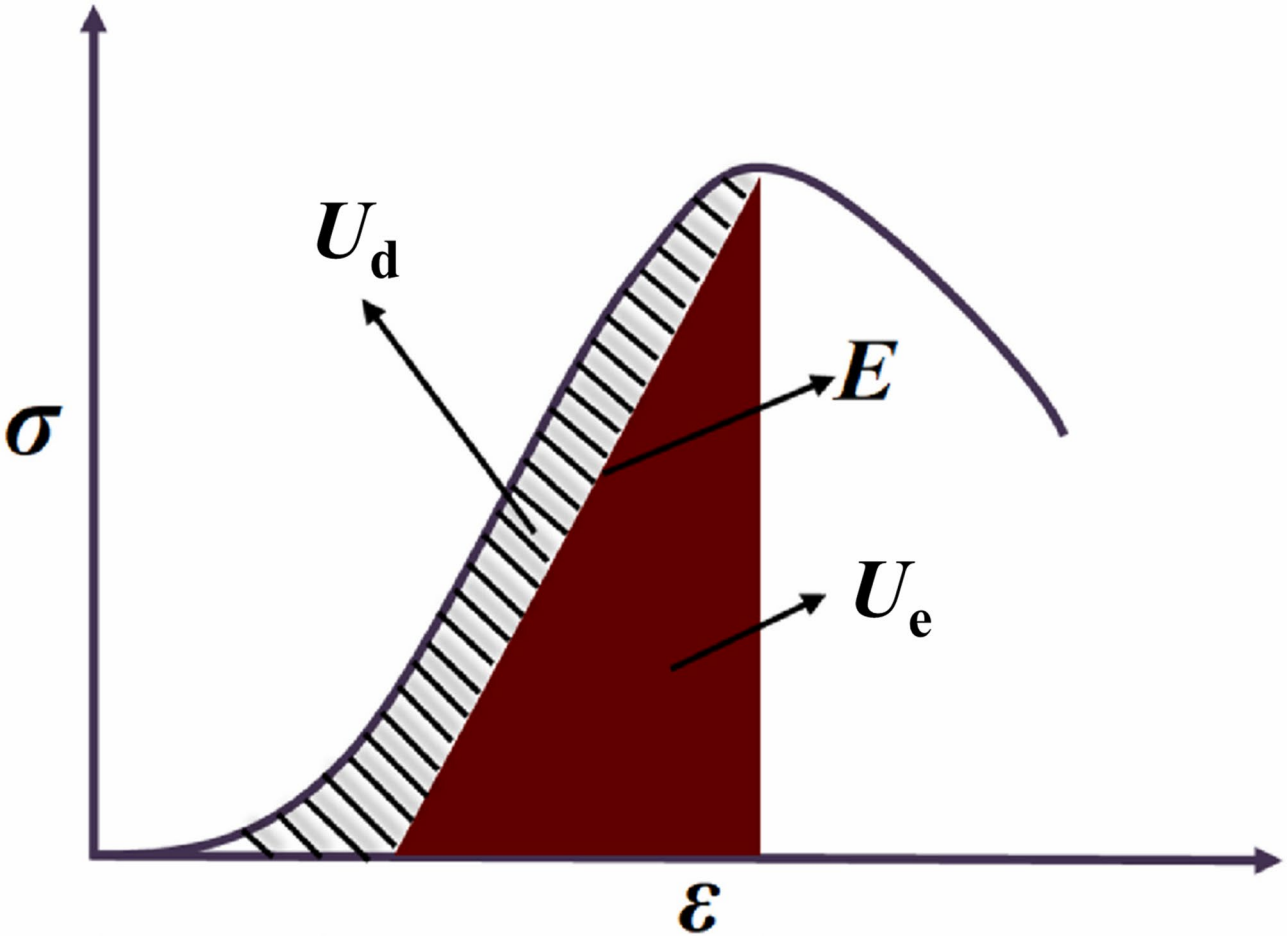
Schematic diagram of strain energy calculation^55^.

As shown in Fig. 5, during the deformation of rock under loading, a portion of the external work is stored as elastic strain energy (*U*_e_), which resists the applied load and is released during unloading. The other part is dissipated as dissipated energy (*U*_d_) through irreversible damage processes such as microcrack initiation, propagation, and frictional slip^52–56^. The total input energy (*U*_in_) can therefore be expressed as:

2 $$U_{{{\text{in}}}} = U_{{\text{e}}} + U_{{\text{d}}}$$

On the stress–strain curve, the total strain energy density (energy per unit volume) corresponding to a given strain *ε* is:

3 $$U_{{{\text{in}}}} = \int_{0}^{\varepsilon } \sigma {\text{d}}\varepsilon$$

where *σ* is the axial stress, MPa; and *ε* is the axial strain.

The elastic strain energy density can be estimated from the slope of the pre-peak unloading curve as:

4 $$U_{{\text{e}}} = \frac{{\sigma^{2} }}{2E}$$

where *E* is the elastic modulus, MPa.

In practical calculations, the total input energy *U*_in_ is obtained by integrating the experimental stress–strain data. The elastic strain energy *U*_e_ is determined using the elastic modulus derived from regression of the pre-peak linear segment, and the dissipated energy *U*_d_ is then determined accordingly. This energy decomposition method quantitatively characterizes the rock’s capacity for energy storage and the extent of energy dissipation associated with damage, thereby providing valuable insights into the mechanisms of damage evolution^57–62^.

**Fig. 6:**
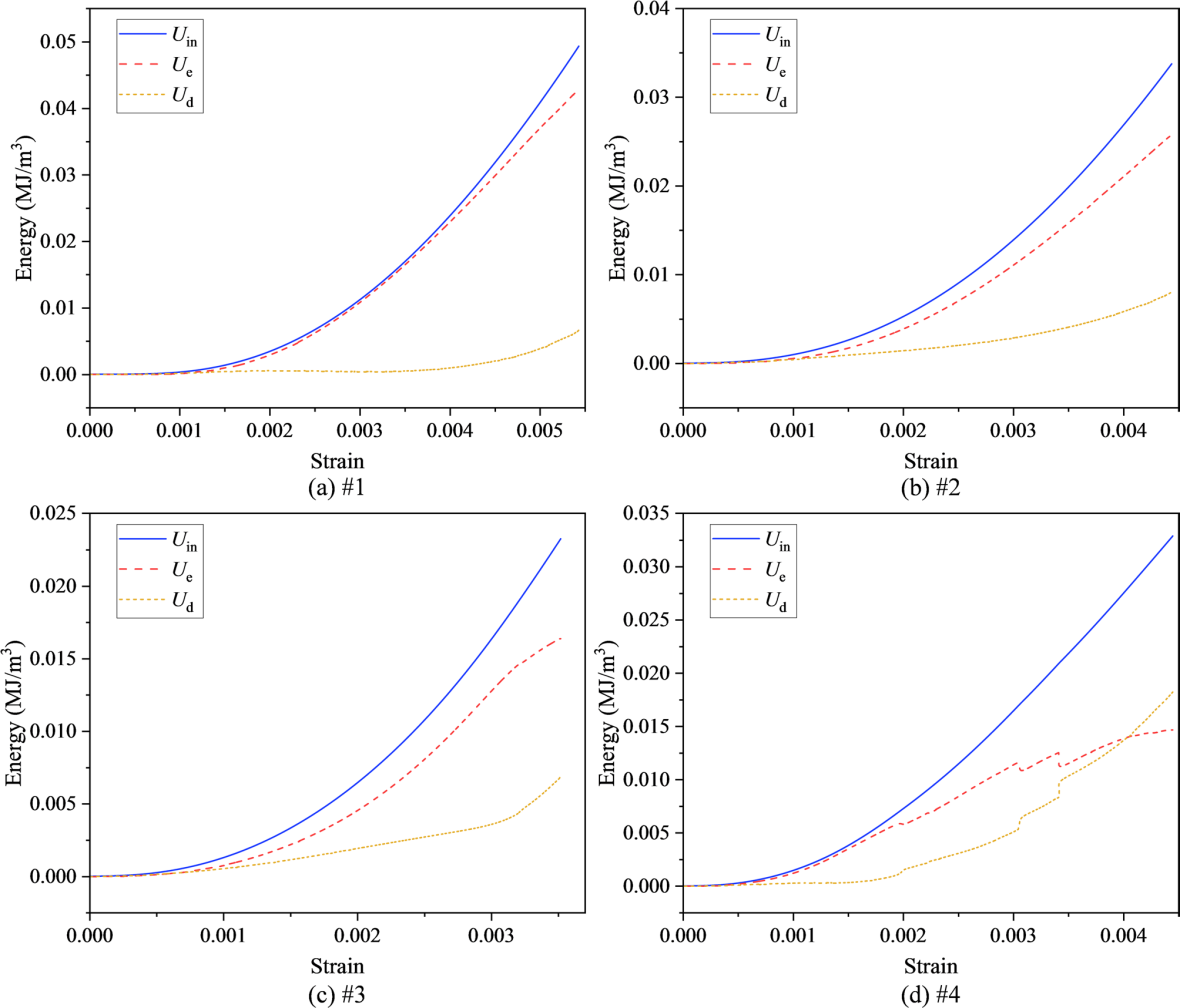
Strain energy evolution curves of damaged mudstone. (**a**) #1; (**b**) #2; (**c**) #3; (**d**) #4.

As shown in Fig. 6, a systematic analysis of the strain energy evolution across the four specimen groups indicates that, at small strains, all groups display an elastic response in which the input energy and elastic energy nearly coincide, while the dissipated energy remains close to zero. With increasing strain, the elastic energy begins to deviate significantly from the input energy, and the dissipated energy changes from near zero to a monotonically increasing trend. This transition signifies the shift from reversible deformation to irreversible damage. This “energy inflection point” appears earliest in the high-damage specimen (#4), followed by #3 and #2, and latest in the undamaged specimen (#1), demonstrating that initial damage significantly advances the onset of damage evolution.

From the energy distribution before the peak, the total input energies of the four groups were 0.0493, 0.0337, 0.0232, and 0.0328 MJ/m^3^; the corresponding elastic energies were 0.0427, 0.0257, 0.0164, and 0.0147 MJ/m^3^; and the dissipated energies were 0.0066, 0.0080, 0.0069, and 0.0182 MJ/m^3^, respectively. As the damage level increases, the proportion of elastic energy gradually decreases, indicating a reduced capacity for energy storage and increased energy dissipation through microcrack propagation and frictional slip. In contrast, the high-damage group (#4) exhibited an earlier and more rapid increase in dissipated energy, with step-like surges in the medium-strain stage that reflect cascading crack-cluster propagation and localized coalescence. The medium-damage group (#3) showed a more gradual increase in dissipated energy, followed by acceleration in the later stage. The low-damage group (#2) and undamaged group (#1) only showed substantial increases in dissipated energy near the peak. This pattern aligns closely with the mechanical behavior illustrated in Fig. 2, where higher damage levels correspond to an earlier onset of the pre-peak nonlinear stage and more pronounced stress drops.

### Dissipated energy ratio analysis

In energy analysis, the dissipated energy ratio (*η*_d_) is employed to quantify the proportion of external input energy transformed into irreversible damage within the material, thereby reflecting the intensity of crack evolution and energy dissipation behavior. It is defined as:

5 $$\eta_{{\text{d}}} = \frac{{U_{{\text{d}}} }}{{U_{{{\text{in}}}} }} \times 100\%$$

At the initial loading stage, cracks within the specimen remain inactive, and *η*_d_ remains close to zero. With increasing strain, crack initiation and propagation occur, resulting in a rapid increase in dissipated energy and a gradual rise in *η*_d_. When the specimen enters the pre-peak nonlinear stage, the growth rate of *η*_d_ increases significantly and reaches its maximum near the peak, indicating that energy partitioning shifts progressively from being dominated by elastic storage to being dominated by dissipation. Thus, *η*_d_ serves as a valuable quantitative indicator of damage evolution, providing an energy-based criterion for identifying precursors to rock instability.

**Fig. 7:**
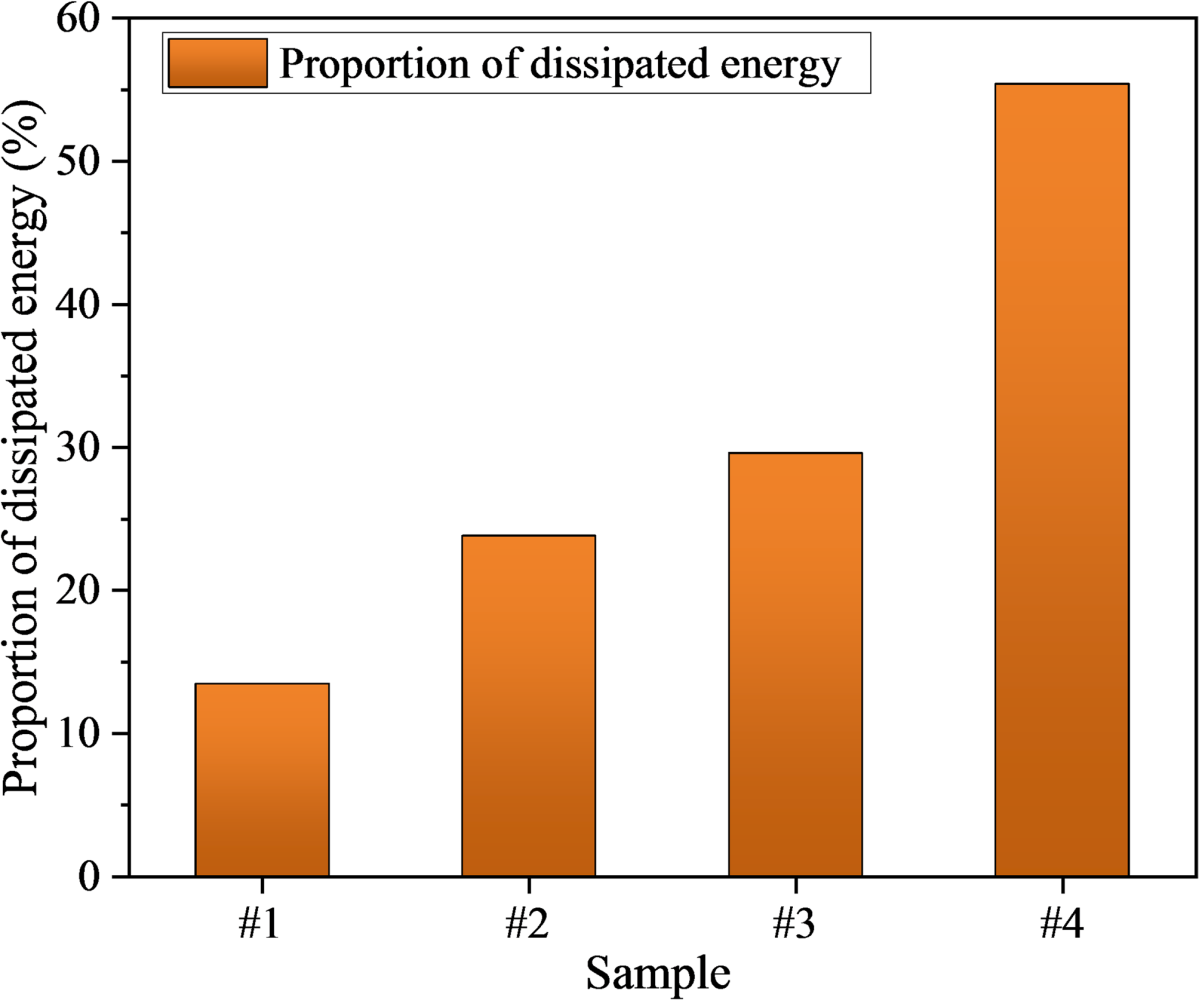
Dissipated energy ratio of damaged mudstone.

As shown in Fig. 7, the dissipated energy ratio of damaged mudstone specimens increases significantly with rising damage levels. It rises from 13.45% in the undamaged group to 23.80% in the low-damage group and 29.59% in the medium-damage group, before surging to 55.42% in the high-damage group, exhibiting a “gradual increase–sharp rise” trend. This pattern indicates that once the damage level surpasses a critical threshold, energy partitioning shifts rapidly from elastic storage dominance to dissipation dominance. This trend is highly consistent with the peak strength reduction in Fig. 3 and the elastic modulus degradation in Fig. 4. The weakening of energy storage capacity reduces the amount of elastic energy that can be stored at the same strain, resulting in a larger portion of the input energy being dissipated by irreversible processes such as crack propagation and frictional slip, as reflected by the increase in dissipated energy ratio. In particular, the increase from 13.45% to 29.59% is relatively gradual, whereas the sharp rise from 29.59% to 55.42% reflects a transition in the medium-to-high damage range from dispersed crack initiation to clustered, cooperative propagation and eventual localized coalescence. This transition leads to a pronounced increase in dissipated energy and its proportion.

### Evolution characteristics of AE counts

AE technology is a non-destructive testing method based on passive monitoring principles. By recording transient elastic waves generated during material deformation and failure, AE reflects the dynamic processes of microcrack initiation, propagation, and coalescence within the material^63–65^. When local stress concentrations exceed the strength limit, microfracturing or frictional slip occurs, releasing energy in the form of stress waves. These waves are detected by piezoelectric sensors attached to the specimen surface, converted into electrical signals, and subsequently processed through preamplification, filtering, and data acquisition systems to extract multiple characteristic parameters, including amplitude, rise time, duration, counts, and energy^66–68^. Compared with macroscopic mechanical indicators such as stress–strain curves, AE technology can capture early microcrack activity prior to macroscopic instability, offering the advantages of high sensitivity, accurate localization, and real-time monitoring. As a result, it has been widely applied in rock mechanics, structural health monitoring, and disaster early warning.

**Fig. 8:**
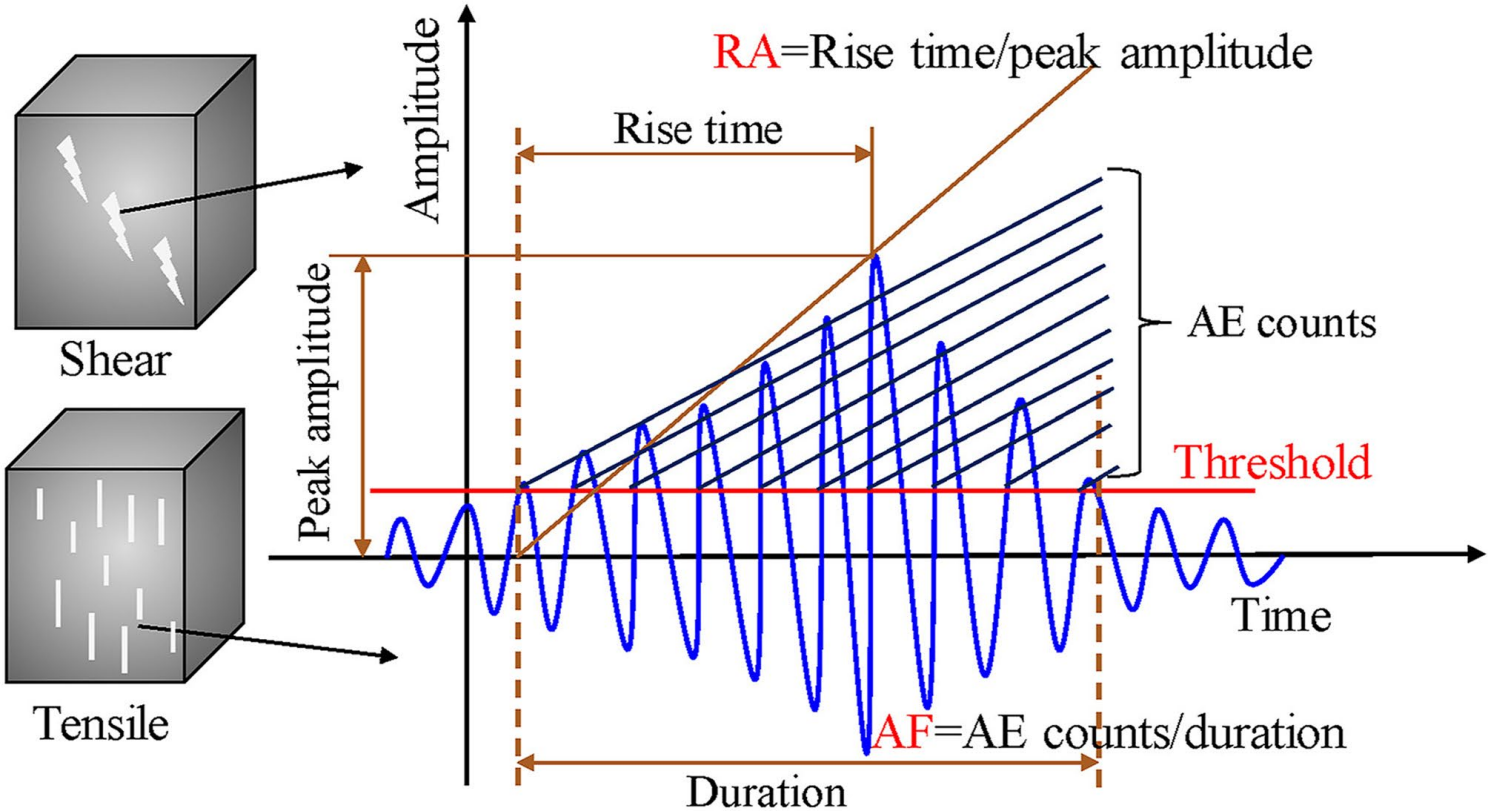
Schematic diagram of AE parameters^67^.

As shown in Fig. 8, AE counts represent the number of times the AE signal amplitude exceeds a preset threshold during its duration, characterizing the intensity and frequency of microcrack activity. The RA (rise angle) value, defined as the ratio of the rise time to the peak amplitude, can be used to differentiate crack failure types. The AF (average frequency) value, calculated as the ratio of counts to duration, reflects the frequency characteristics of the signal. By applying the combined RA–AF criterion, AE signals can be effectively classified according to their associated fracture modes. When integrated with the time-series evolution of AE parameters and energy characteristics, this approach provides deeper insight into crack evolution mechanisms and damage development patterns in materials under loading.

**Fig. 9:**
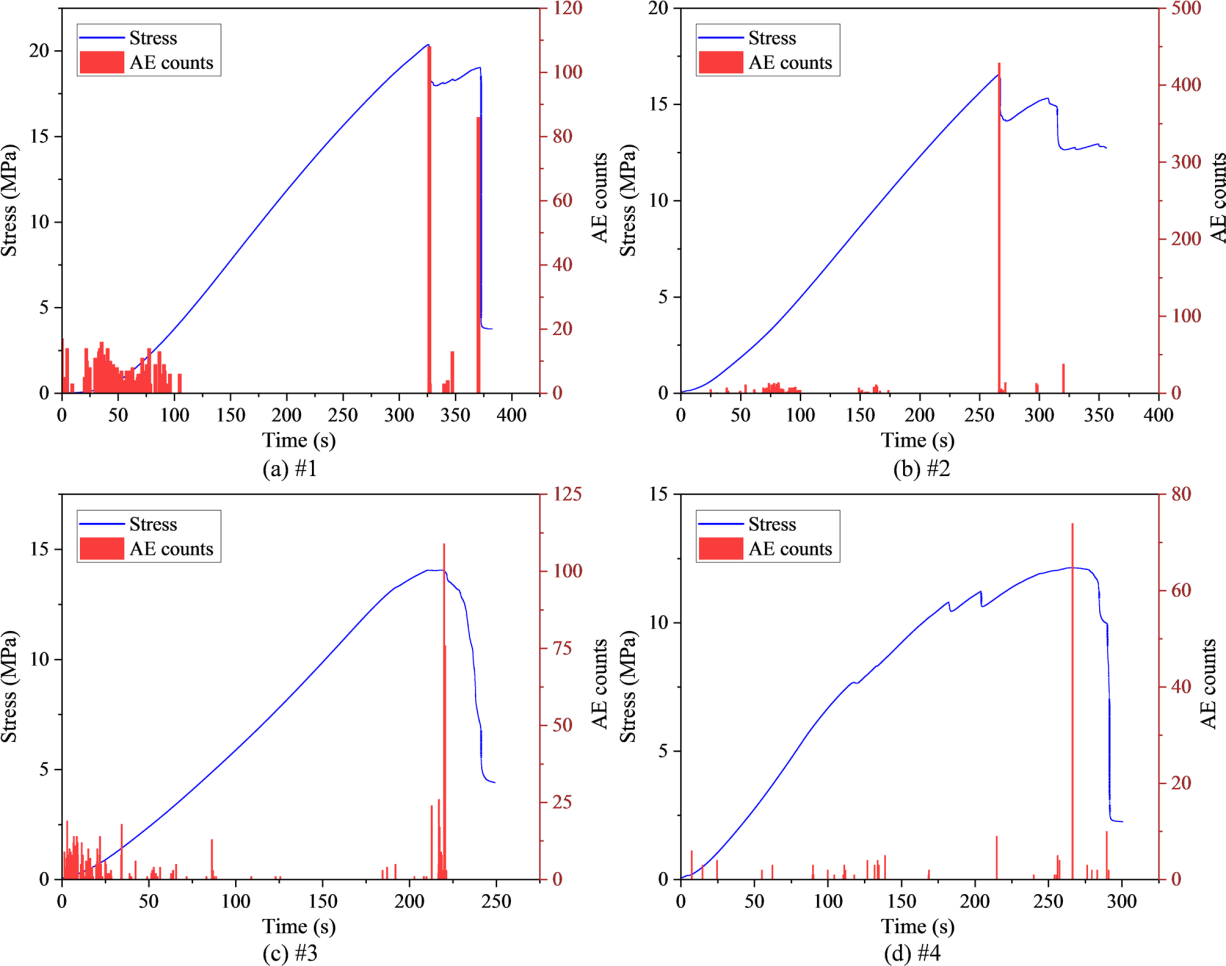
AE count characteristics of damaged mudstone. (**a**) #1; (**b**) #2; (**c**) #3; (**d**) #4.

Figure 9 presents the AE count evolution characteristics of mudstone specimens with different damage levels. For the undamaged specimen (#1), only a few sporadic counts associated with pore closure appear at the early loading stage, followed by a long period of low activity until a concentrated high-amplitude surge occurs just before the peak strength. After the peak, the counts decrease rapidly as load-bearing capacity collapses. This behavior reflects the suppression of microcrack initiation in an intact structure, with unstable propagation occurring mainly near the ultimate strength. In the low-damage specimen (#2), AE activation initiates at a lower stress level compared with specimen #1, and intermittent small bursts occur prior to the peak. However, the dominant surge still appears near the peak, indicating that under light damage, cracks begin to activate at lower stress levels, but overall failure is still dominated by a single main rupture event. For the medium-damage specimen (#3), AE activity becomes pronounced at intermediate stress levels, with multiple step-like surges occurring before the peak, followed by the main rupture. These multi-stage surges correspond to successive instabilities and local coalescence of crack clusters, demonstrating that the onset of the unstable growth stage is advanced considerably. It is noteworthy that the highly damaged specimen (#4) exhibited lower AE counts in the initial compaction stage compared to the medium-damage specimen (#3). This can be attributed to the nature of the pre-existing damage. The high preload (60% UCS) likely generated larger cracks that closed gradually through less audible mechanisms like frictional slip, resulting in suppressed early AE activity. The intense AE response was delayed until the stage where new cracks initiated from these large flaws and coalesced, leading to the observed sharp surges.

## Discussion

### RA–AF characteristics

The AE count evolution during loading has been described in Section "Evolution characteristics of AE counts", showing clear trends of earlier activation and increased precursor activity with damage level. Building on these observations, this section further explores the underlying fracture mechanisms using advanced AE parameters to reveal the progressive deterioration behavior of damaged mudstone.

The RA-AF criterion is commonly used to distinguish fracture types of AE events^68^. RA is defined as the ratio of rise time to peak amplitude, reflecting the combined characteristic of the signal’s rise rate and amplitude, while AF is defined as the ratio of counts to duration. When AE events are projected onto the RA–AF plane, tensile cracks tend to occupy the “low RA–high AF” region (upper left), whereas shear cracks are typically located in the “high RA–low AF” region (lower right). The separation boundary can be determined empirically or using data-driven approaches^69–72^. In this study, an empirical threshold of AF/RA = 50 was adopted. By calculating the temporal proportions of tensile and shear cracks, fracture mechanism transitions and precursors to instability can be effectively identified. This classification is based on the understanding that tensile cracks typically produce sharper waveforms with higher frequencies, whereas shear cracks generate longer-duration signals with lower frequency due to more frictional slip. While this method is semi-empirical and subject to material-specific variations, it remains a practical and robust approach when combined with cumulative statistical trends, as adopted in this study.

**Fig. 10:**
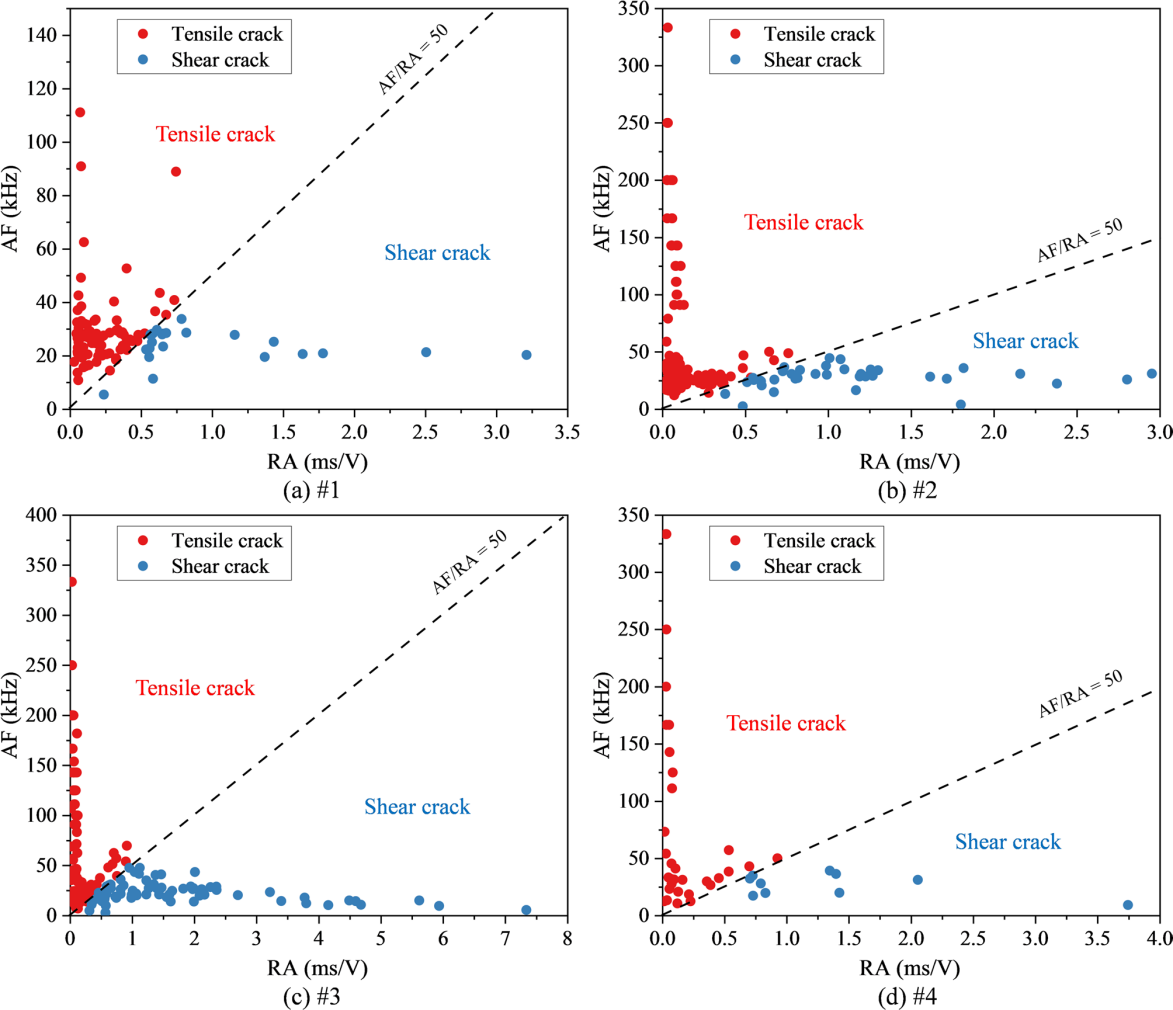
RA–AF characteristics of damaged mudstone. (**a**) #1; (**b**) #2; (**c**) #3; (**d**) #4.

**Fig. 11:**
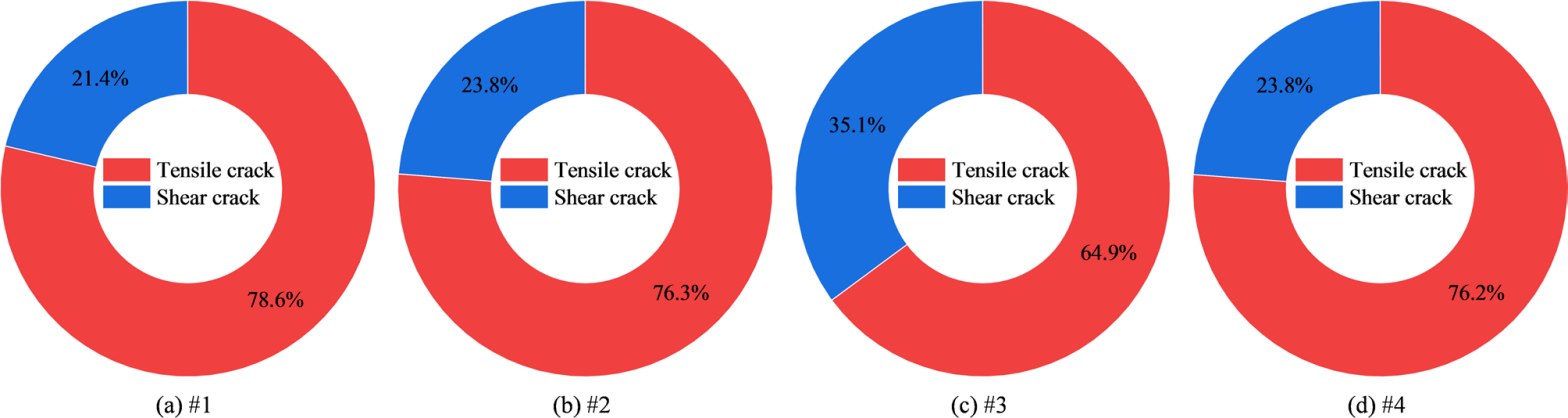
Proportions of tensile and shear cracks in damaged mudstone. (**a**) #1; (**b**) #2; (**c**) #3; (**d**) #4.

Figure 10 illustrates the RA–AF characteristics of mudstone specimens with different damage levels, while Fig. 11 presents the corresponding proportions of tensile and shear cracks. The tensile crack proportions were 78.64%, 76.25%, 64.90%, and 76.19%, respectively, while the shear crack proportions were 21.36%, 23.75%, 35.10%, and 23.81%. These results demonstrate that tensile failure remains the dominant mode in damaged mudstone. However, with increasing damage levels, the proportion of shear failure gradually rises, indicating a shift toward mixed-mode failure, where tensile cracking remains dominant but shear contribution increases significantly.

Predamage influences the composition of AE events, expressed as the relative proportions of tensile and shear signals derived from RA–AF classification. This effect arises because predamage increases porosity and crack density of the specimen while reducing the contact stiffness among grains, matrix, and bedding planes. As a result, AE signals exhibit longer rise times and extended durations (higher RA, lower AF), promoting a transition in cracking behavior from dispersed tensile opening to clustered shear-slip and localized coalescence. This trend is highly consistent with the strength degradation in Fig. 2 and the sharp increase in dissipated energy ratio in Fig. 7.

### AE *b*-value and* S*-value

The AE *b*-value is commonly used to characterize the steepness of the size distribution of AE events. It is derived from the frequency–magnitude relationship of AE signals, analogous to the Gutenberg–Richter relation in seismology, and reflects the relative proportion of high-energy to low-energy events within a given time window. The *b*-value can be calculated by the following Eq. (6)^73^.

6 $${\text{l}} {\text{o}}g_{10} N = a - bM$$

where *N* denotes the accumulated AE frequency within the magnitude range; a refers to an experimentally derived parameter. *M* represents the magnitude of the AE event, and is calculated based on the measured AE amplitude using the expression: A/20^74,75^.

A smaller *b*-value corresponds to a flatter distribution, indicating a higher proportion of large-amplitude, high-energy events. This typically reflects the presence of larger effective crack sizes, enhanced clustered propagation, and stronger localization, all of which suggest an elevated risk of instability. Conversely, a larger *b*-value indicates a predominance of small events, crack initiation mainly in a dispersed manner, and relatively stable structural conditions.

**Fig. 12:**
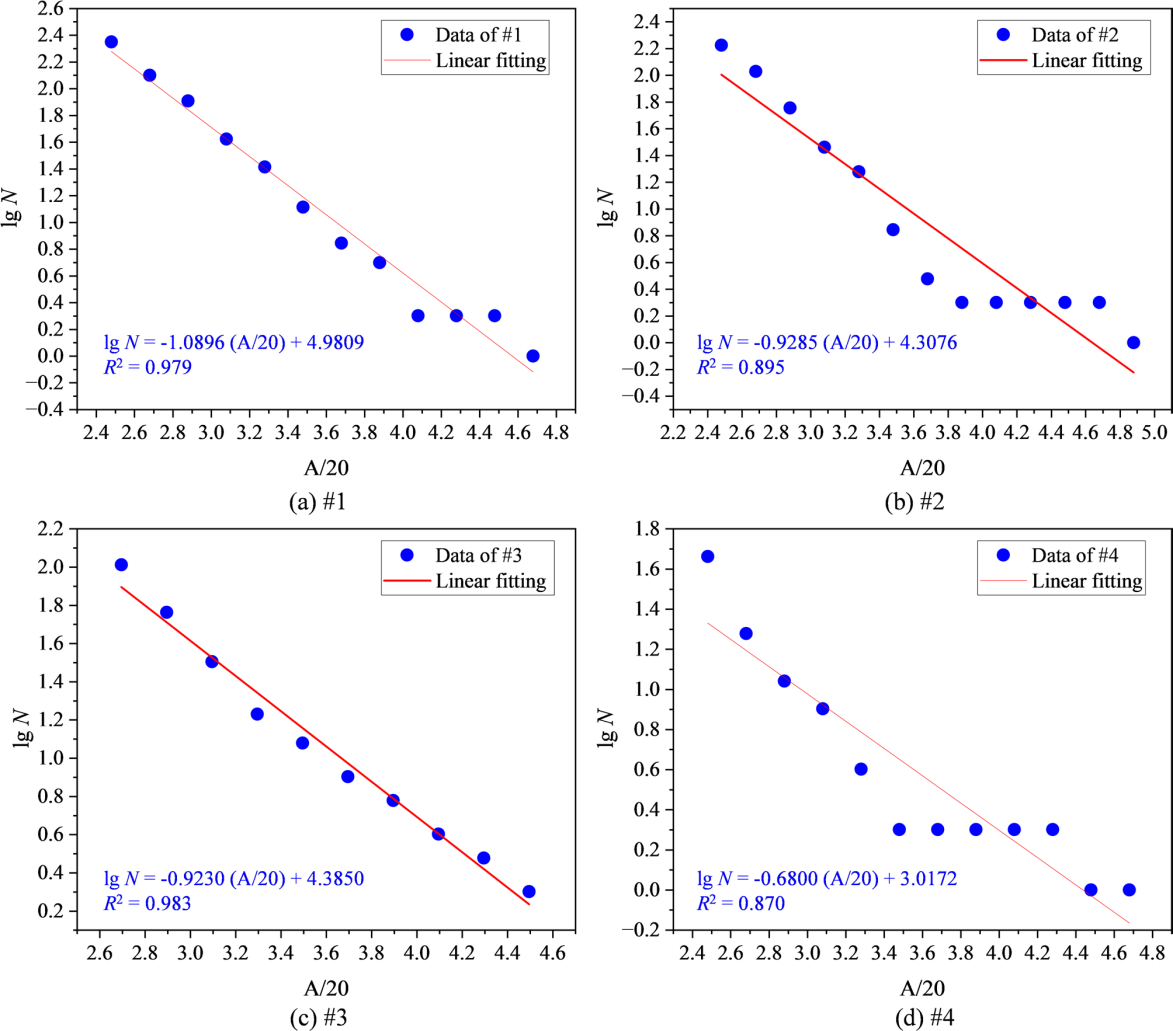
AE *b*-value fitting process for damaged mudstone (the blue circles represent the cumulative frequency-magnitude distribution for each sample, the solid red line is the linear regression fit, and the slope of which is the *b*-value.). (**a**) #1; (**b**) #2; (**c**) #3; (**d**) #4.

**Fig. 13:**
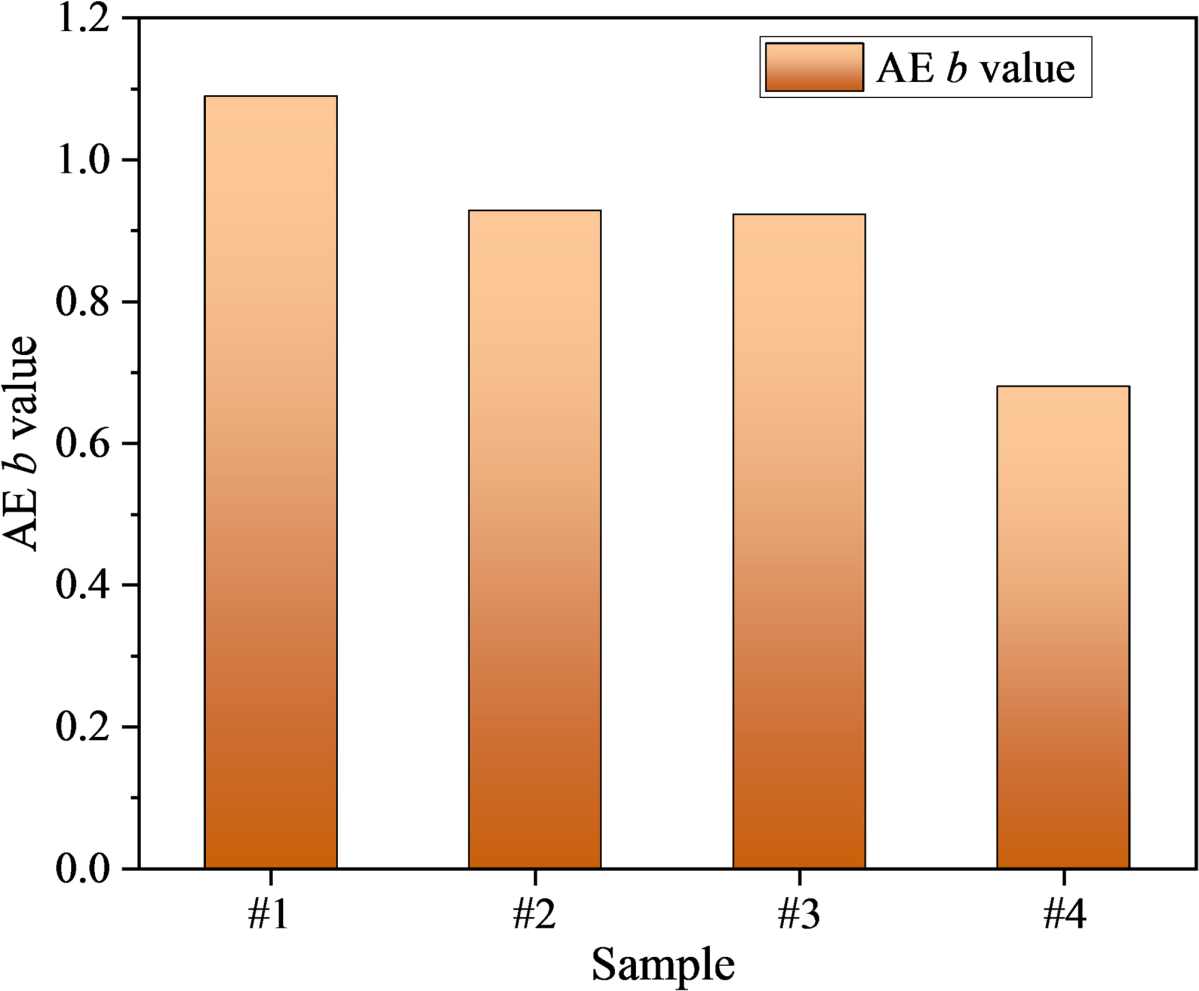
Variation of AE *b*-value in damaged mudstone.

In this study, AE amplitude data over the entire loading stage were grouped with a bin size of 4 dB for frequency–magnitude statistical analysis. The results were linearly fitted in semi-logarithmic coordinates using the least-squares method. As shown in Fig. 12, the fitted lines and scatter plots for each damage group exhibit good correlation. Figure 13 further compares the *b*-values of mudstone specimens at different damage levels, which were 1.089, 0.929, 0.923, and 0.680, respectively. The undamaged group (#1) exhibited the highest *b*-value, indicating that small events predominated, crack growth occurred primarily through dispersed opening, and structural integrity remained relatively high. In the low- and medium-damage groups (#2 and #3), the *b*-value decreased, suggesting a higher proportion of large events and that frictional slip and clustered propagation began to dominate damage accumulation. The high-damage group (#4) had the lowest *b*-value, indicating that a limited number of high-energy shear events were sufficient to trigger localized coalescence, representing the highest instability risk.

This trend is highly consistent with the increased shear crack proportion in Fig. 11 and the elevated dissipated energy ratio in Fig. 7, indicating that predamage drives a shift in energy partitioning from elastic storage dominance to dissipation dominance.

In addition, the AE activity *S*-value reflects the concentration of event scales in terms of energy release and is commonly used to assess the relative dominance of large- versus small-amplitude AE events. A higher *S*-value indicates that AE activity is controlled by a small number of high-energy events, suggesting the formation of large-scale cracks. The calculation process of the AE *S*-value is given in Eq. (7)^76,77^.

7 $$S = 0.117\lg (n + 1) + 0.029\lg \frac{1}{N}\sum\limits_{i = 1}^{n} 1 0^{{0.075M_{i} }} + 0.00075M_{\max }$$

where *S* represents a constant; *M*_max_ represents the AE maximum magnitude;* M*_*i*_ represents the AE magnitude.

The *S*-values of mudstone specimens with different damage levels were 0.202, 0.243, 0.267, and 0.281, respectively. Unlike the *b*-value, the *S*-value increased with higher damage levels. An increase in *S*-value indicates that AE activity within the statistical window becomes more concentrated, with higher average energy levels and stronger extreme events, signifying a transition in cracking behavior from dispersed tensile opening to clustered cooperative propagation and shear-dominated localization. In particular, the medium-damage specimen (#3) exhibited a continued rise in *S*-value accompanied by the peak proportion of shear cracks, indicating entry into the unstable growth domain. The high-damage specimen (#4) displayed only a slight further increase in *S*-value yet reached the highest level overall, reflecting that a limited number of high-energy shear events dominated and ultimately drove failure coalescence.

This evolutionary trend is consistent with the enhanced shear characteristics identified by RA–AF analysis, the reduction in *b*-value, and the increase in dissipated energy ratio, confirming that predamage shifts the energy distribution from elastic storage dominance toward dissipation dominance and progressively drives the system closer to the instability threshold.

### Mechanical deterioration mechanism of damaged mudstone

By integrating the stress–strain response, energy partitioning, and multi-parameter AE results, the deterioration process of mudstone subjected to preloading and unloading damage under uniaxial compression can be summarized as a progressive evolution from elastic dominance → damage activation → shear localization. The preloading and unloading damage treatment induces irreversible tensile openings and micro-shear slip along bedding planes and grain–matrix interfaces, thereby reducing the effective load-bearing area and contact stiffness. Consequently, both the elastic modulus and peak strength decrease monotonically, with accelerated degradation observed in the high-damage range (Figs. 2, 3 and 4).

From an energy perspective, at low damage levels the total input energy and elastic strain energy are nearly identical, while dissipated energy remains close to zero. With increasing damage levels, the “energy inflection point” shifts toward lower strain, and both dissipated energy and its proportion increase markedly—from 13.45 to 55.42% (Fig. 7). This indicates that a larger fraction of the input energy is consumed earlier by microcrack propagation and frictional slip.

**Fig. 14:**
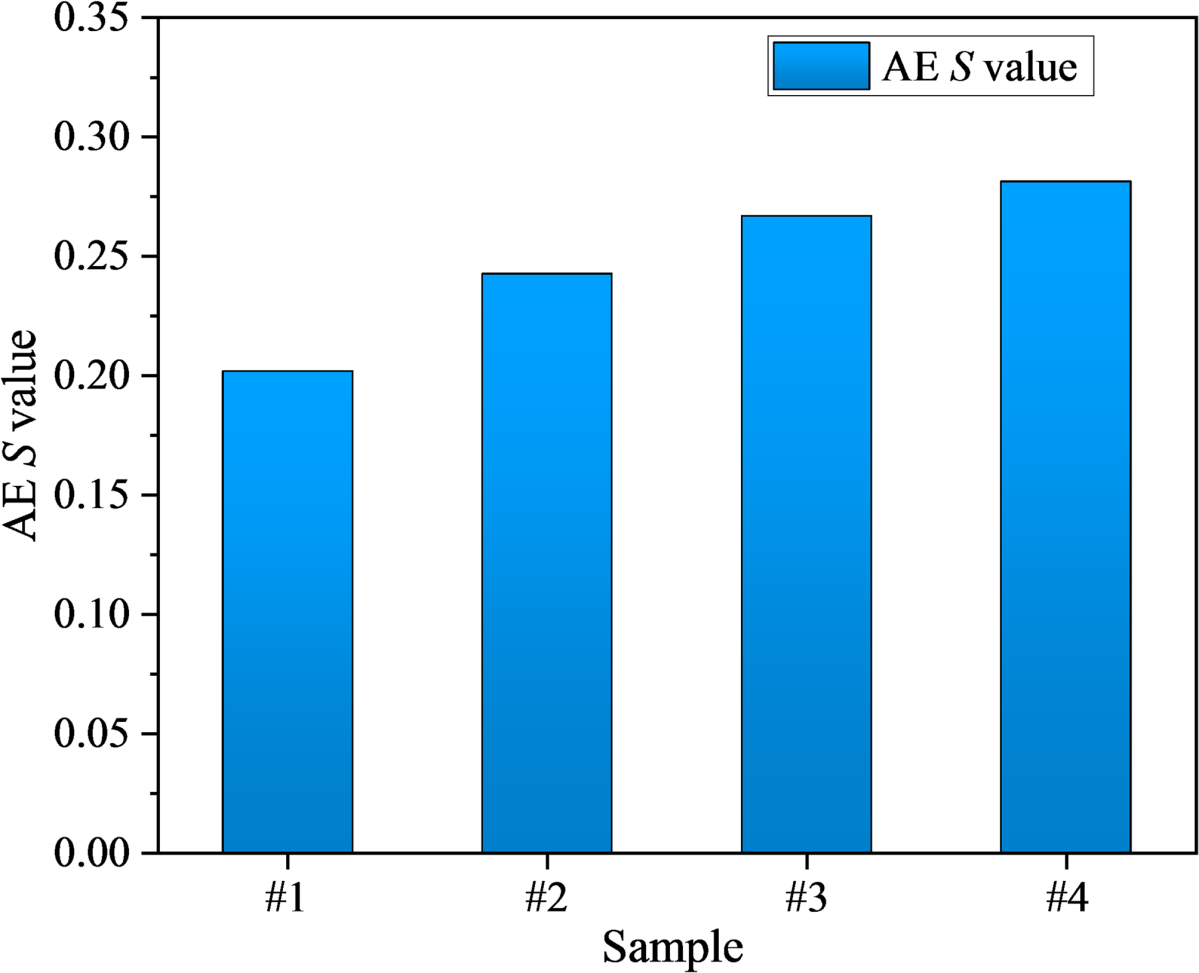
Variation of AE *S*-value in damaged mudstone.

Corresponding AE behavior also exhibits systematic changes. AE counts are activated earlier and display step-like surges prior to peak stress (Fig. 9). RA–AF analysis reveals a gradual shift from tensile-dominated cracking to cooperative shear involvement. In addition, the AE *b*-value decreases from 1.089 to 0.680, indicating a rising proportion of large-amplitude, high-energy events (Fig. 13), while the AE *S*-value increases from 0.202 to 0.281, reflecting a continuous rise in event concentration, mean energy level, and extreme event intensity (Fig. 14). Collectively, these results demonstrate that predamage shifts energy partitioning from elastic storage to dissipation dominance, promotes cooperative crack-cluster propagation and localization through shear slip along bedding planes, and ultimately accelerates brittle instability.

The degradation behavior observed under cyclic loading conditions has clear engineering implications. In underground mining and tunneling operations, particularly in stratified soft rock environments, the rapid reduction in elastic modulus and increase in AE activity can serve as early indicators of structural weakening. For instance, a transition in AE b-value trends may signal a shift toward unstable crack propagation, warranting preemptive reinforcement or evacuation. Moreover, the established correlation between damage levels and AE-derived crack classifications provides a potential foundation for in-situ real-time stability monitoring, enabling risk-based decision-making during excavation or long-term operation.

While this study provides valuable insights into the degradation behavior and AE characteristics of mudstone under cyclic loading, several limitations must be acknowledged. First, the results are based on a specific type of mudstone with particular mineralogical composition and structural features, which may not be representative of all soft rocks. Second, the cyclic loading was performed at a constant rate and stress amplitude range; variations in loading frequency, waveform, and confining pressure conditions could alter the observed damage evolution. Therefore, the findings should be interpreted within the defined experimental framework, and caution should be exercised when extending these results to different geological settings or loading conditions. Future studies should explore the effects of loading frequency, environmental conditions (e.g., water content), and scale effects to improve the generalizability of the conclusions.

## Conclusions

This study systematically investigated the mechanical degradation behavior and AE characteristics of mudstone under different levels of initial damage and cyclic loading conditions. The main conclusions are as follows:

1. The average peak strength of damaged mudstone decreases with increasing damage levels, with values of 20.339 MPa, 16.608 MPa, 13.775 MPa and 12.241 MPa. The elastic modulus also decreases, with values of 4.861, 4.645, 4.435, and 3.871 GPa. An “accelerated reduction” is observed in the 40–60% UCS predamage range, indicating a transition from slow microcrack initiation to localized coalescence.
2. The evolution of strain energy reveals that the energy inflection point shifts to earlier stages with greater damage, whereas the total input energy and elastic energy before the peak decrease significantly. In contrast, dissipated energy and its proportion increase markedly, with the dissipated energy ratio increasing from 13.45 to 55.42%. The high-damage group exhibits step-like surges, corresponding to cascading crack-cluster propagation and localized coalescence.
3. Higher damage levels result in earlier AE activation and more distinct pre-peak step-like bursts. The RA–AF results indicate that tensile failure remains dominant in damaged mudstone; however, the proportion of shear failure increases progressively with increasing damage level. This finding suggests that as the microcrack density and bedding plane weakness increase, frictional slip and shear propagation shift from a cooperative role to a dominant role.
4. With increasing damage levels, the *b*-value decreases from 1.089 to 0.680, indicating a higher proportion of high-energy events, while the AE *S*-value increases from 0.202 to 0.281, reflecting concurrent rises in event concentration, average energy level, and extreme event intensity. The observed decrease in *b*-value and increase in *S*-value are consistent with the trends in RA–AF and dissipated energy ratio, together forming a multi-source evidence chain for the progressive deterioration of damaged mudstone.

## Data Availability

All relevant data are within the manuscript.

## References

[1] Chang, X. Y., Liang, Y. P. & Ran, Q. C. Mechanical response of mudstone based on acoustic emission fractal features. *Fractal Fract.* **9**, 83. 10.3390/fractalfract9020083 (2025).

[2] Zhao, J. J. et al. Microscopic mechanism of wettability alteration at the coal-water-CO2 interface under extreme conditions. *Phys. Fluids* **37**, 072020. 10.1063/5.0276577 (2025).

[3] Zou, Q. L. et al. Effect of particle size and dosage on hydration kinetics of nano-alumina-modified cement. *Constr. Build. Mater.* **494**, 143283. 10.1016/j.conbuildmat.2025.143283 (2025).

[4] Zhao, J. J. et al. Effect of silica nanofluid on coal wettability and its stability characterization. *Phys. Fluids* **37**, 022015. 10.1063/5.0253229 (2025).

[5] Liang, Y. P., Sun, W. J., Wu, Z. P., Mao, S. R. & Ran, Q. C. Effect of disturbed coal pore structure on gas adsorption characteristics: Mercury intrusion porosimetry. *Front. Energy Res.* **12**, 1333686. 10.3389/fenrg.2024.1333686 (2024).

[6] He, Y. Y. et al. Constitutive model for Ya’an mudstone based on mesoscopic breakage mechanism. *J. Mt. Sci.* **20**, 1159–1169. 10.1007/s11629-022-7630-0 (2023).

[7] Zou, J. P. et al. Experimental study on the mechanical characteristics of weakly cemented mudstone under different loading rates. *Sci. Rep-Uk* **14**, 15364. 10.1038/s41598-024-65024-1 (2024). 10.1038/s41598-024-65024-1 PMC11224246 38965259

[8] Liang, Y. P. et al. Characteristics of overburden fracture conductivity in the shallow buried close coal seam group and its effect on low-oxygen phenomena. *Phys. Fluids* **37**, 047103. 10.1063/5.0260321 (2025).

[9] Zhao, J. J. et al. Mechanism of water adsorption and penetration at the coal interface under methane pressure and temperature. *Phys. Fluids* **37**, 062018. 10.1063/5.0273416 (2025).

[10] Sun, D. L., Liang, Y. P., Huang, X. C. & Ran, Q. C. Progress and prospects of coalbed methane development and utilization in coal mining areas with large dip angle and multiple coal groups in Xinjiang. *Coal Sci. Technol.* **51**, 161–171. 10.13199/j.cnki.cst.mcq2022-1325 (2023).

[11] Zhang, T. C. et al. Impact of gas adsorption on coal relative permeability: a laboratory study. *Int. J. Rock Mech. Min. Sci.* **194**, 106191. 10.1016/j.ijrmms.2025.106191 (2025).

[12] Huang, K. et al. Mechanical behavior and fracture mechanism of red-bed mudstone under varied dry-wet cycling and prefabricated fracture planes with different loading angles. *Theoret. Appl. Fract. Mech.* **127**, 104094. 10.1016/j.tafmec.2023.104094 (2023).

[13] Sun, X. Y., Ran, Q. C., Liu, H., Ning, Y. H. & Ma, T. F. Characteristics of stress-displacement-fracture multi-field evolution around gas extraction borehole. *Energies* **16**, 2896. 10.3390/en16062896 (2023).

[14] Zhao, J. J. et al. Wettability of the coal–water–Ar interface under coupled temperature and pressure conditions. *Surf. Interfaces* **75**, 107770. 10.1016/j.surfin.2025.107770 (2025).

[15] Zhao, J. J. et al. New insights from molecular dynamic simulation on coal-water interface wettability. *Chem. Eng. Sci.* **318**, 122131. 10.1016/j.ces.2025.122131 (2025).

[16] Moghaddam, R. H. & Golshani, A. Fatigue behavior investigation of artificial rock under cyclic loading by using discrete element method. *Eng. Fail. Anal.* **160**, 108105. 10.1016/j.engfailanal.2024.108105 (2024).

[17] Gu, L. J., Feng, X. T., Kong, R., Yang, C. X. & Xia, Y. L. Evolution of mechanical parameters of Shuangjiangkou granite under different loading cycles and stress paths. *J. Rock Mech. Geotech. Eng.* **16**, 1113–1126. 10.1016/j.jrmge.2023.09.005 (2024).

[18] Tan, H., Song, Y. J., Yang, H. M. & Che, Y. X. Energy evolution and strain localization in fractured sandstone under freeze-thaw cycling and uniaxial loading-unloading. *Int. J. Rock Mech. Min. Sci.* **177**, 105746. 10.1016/j.ijrmms.2024.105746 (2024).

[19] Cui, Z. H. et al. Dynamic fracture properties and criterion of cyclic freeze-thaw treated granite subjected to mixed-mode loading. *J. Rock Mech. Geotechn. Eng.* **16**, 4971–4989. 10.1016/j.jrmge.2023.12.019 (2024).

[20] Ran, Q. C. et al. Deterioration mechanisms of coal mechanical properties under uniaxial multi-level cyclic loading considering initial damage effects. *Int. J. Rock Mech. Min. Sci.* **186**, 106006. 10.1016/j.ijrmms.2024.106006 (2025).

[21] Zhu, J. et al. Fracturing and acoustic emission characteristics of saturated reservoir rocks under constant-amplitude-cyclic loading. *Bull. Eng. Geol. Env.* **83**, 417. 10.1007/s10064-024-03911-7 (2024).

[22] Zhang, Q. H., Meng, X. R. & Zhao, G. M. Energy evolution and fractal characteristics of sandstones under true triaxial cyclic loading and unloading. *Fractal Fract.* **8**, 714. 10.3390/fractalfract8120714 (2024).

[23] Ye, C. F., Xie, H. P., Wu, F. & Li, C. B. Study on the nonlinear time-dependent deformation characteristics and viscoelastic-plastic model of shale under direct shear loading path. *Bull. Eng. Geol. Env.* **82**, 189. 10.1007/s10064-023-03170-y (2023).

[24] Chen, Y. C., Zhao, Z. K. & Guo, J. Precursors of rock failure under cyclic loading and unloading: From the perspective of energy and acoustics. *Eng. Fail. Anal.* **166**, 108860. 10.1016/j.engfailanal.2024.108860 (2024).

[25] Wang, H., Fall, M., Miao, S. J., Yu, S. B. & Shang, X. F. Cyclic loading effects on the strength and fatigue properties of argillaceous siltstone across various characteristic stress intervals. *Eng. Fract. Mech.* **302**, 110059. 10.1016/j.engfracmech.2024.110059 (2024).

[26] Fu, B., Li, Y. C., Tang, C. A., Ji, Y. L. & Zang, A. R. A micromechanical analysis of marble pulverization under quasi-static progressive cyclic loading. *Int. J. Rock Mech. Min. Sci.* **179**, 105786. 10.1016/j.ijrmms.2024.105786 (2024).

[27] Li, S. N. et al. Quantitative calculation of the damage of carbonaceous mudstone during uniaxial compressive failure process under dry-wet cycling. *Bull. Eng. Geol. Env.* **84**, 190. 10.1007/s10064-025-04169-3 (2025).

[28] Zheng, Q. S., Liu, E. L., Yu, D. & Liu, M. X. Fatigue and damage properties of non-consecutive jointed mudstone samples subjected to cyclic triaxial loading. *Bull. Eng. Geol. Env.* **79**, 2467–2481. 10.1007/s10064-019-01693-x (2020).

[29] Li, F. L., Yang, J., Liu, W. Q., Fan, Z. H. & Yang, Y. G. Effect of loading rate change on the mechanical properties of mudstone under uniaxial compression. *Rock Soil Mech.* **42**, 369–378. 10.16285/j.rsm.2020.0846 (2021).

[30] Zhang, D. X. et al. Experimental study on the bearing mechanical characteristics and deformation failure mechanism of damaged sandy mudstone. *Fatigue Fract. Eng. M* **47**, 3204–3219. 10.1111/ffe.14353 (2024).

[31] Li, C. B., Chen, H. J., Ye, C. F., Feng, Z. C. & Ran, Q. C. Acoustic emission spatiotemporal evolution and multifractal spectrum of sandstone under the coupled effects of wet-dry cycles and cyclic loading. *Phys. Fluids* **37**, 087107. 10.1063/5.0278924 (2025).

[32] Ye, C. F. et al. Asymmetric failure mechanisms of anisotropic shale under direct shear. *Int. J. Rock Mech. Min. Sci.* **183**, 105941. 10.1016/j.ijrmms.2024.105941 (2024).

[33] Li, S. Y., Wang, Z. L., Wang, J. G., Yang, J. Q. & Lu, Z. T. Analysis on mechanical behavior and progressive failure of deep-buried marble based on complete stress-strain curves. *Bull. Eng. Geol. Env.* **82**, 133. 10.1007/s10064-023-03123-5 (2023).

[34] Kong, X. G. et al. Time-varying characteristics of acoustic emission and fractals based on information dimension during structural failure of coal subjected to uniaxial compression. *Measurement* **236**, 115088. 10.1016/j.measurement.2024.115088 (2024).

[35] Ran, Q. C. et al. Mechanical behavior and acoustic emission characteristics of initially damaged coal under triaxial cyclic loading and unloading. *J. Rock Mech. Geotech. Eng.*10.1016/j.jrmge.2025.02.009 (2025).

[36] Song, Z. Y. et al. Mechanical responses of sandstone exposed to triaxial differential cyclic loading with distinct unloading rates of confining stress: A lab scale investigation. *Int. J. Coal Sci. Technol.* **12**, 58. 10.1007/s40789-025-00796-z (2025).

[37] Rong, H. Y. et al. Mechanical behaviour and acoustic emission characteristics of the reinforced soft muddy rock under various moisture content levels. *Int. J. Coal Sci. Technol.* **11**, 84. 10.1007/s40789-024-00733-6 (2024).

[38] Wei, M. H. et al. Acoustic and electromagnetic emission laws of rocks affected by size-dependent failure behavior under Brazilian tests. *Eng. Fail. Anal.* **167**, 109005. 10.1016/j.engfailanal.2024.109005 (2025).

[39] Fu, Y. K. et al. Mechanical properties and energy evolutions of burst-prone coal samples with holes and fillings. *Int. J. Coal Sci. Technol.* **11**, 40. 10.1007/s40789-024-00675-z (2024).

[40] Liu, B., Liu, Y., Xiao, P. W. & Zhang, L. Effects of a single flaw on failure and acoustic emission characteristics around circular opening subjected to biaxial compression. *Eng. Fail. Anal.*10.1016/j.engfailanal.2024.109008 (2025).

[41] Jiang, J. D. & Xu, J. Investigation of energy mechanism and acoustic emission characteristics of mudstone with different moisture contents. *Shock. Vib.* **2018**, 2129639. 10.1155/2018/2129639 (2018).

[42] Meng, Q. B., Zhang, M. W., Han, L. J., Pu, H. & Nie, T. Y. Effects of acoustic emission and energy evolution of rock specimens under the uniaxial cyclic loading and unloading compression. *Rock Mech. Rock Eng.* **49**, 3873–3886. 10.1007/s00603-016-1077-y (2016).

[43] Hou, R. B., Zhang, K., Tao, J., Xue, X. R. & Chen, Y. L. A nonlinear creep damage coupled model for rock considering the effect of initial damage. *Rock Mech. Rock Eng.* **52**, 1275–1285. 10.1007/s00603-018-1626-7 (2019).

[44] Huang, P., Zhang, J. X., Damascene, N. J., Wang, Z. J. & Li, M. Effect of loading rate on mechanical behavior of coal samples with initial damage accumulation. *Mech. Time-Depend. Mater.* **26**, 309–322. 10.1007/s11043-021-09489-x (2022).

[45] Yu, W. X., Jin, L., Du, X. L. & Deng, X. F. Effect of initial damage state on static and dynamic fracture of concrete with different sizes: An experimental study. *Eng. Fract. Mech.* **274**, 108797. 10.1016/j.engfracmech.2022.108797 (2022).

[46] Lu, A. H., Xu, J. H., Xia, Y. & Sun, L. Study on dynamic behavior and energy dissipation of rock considering initial damage effect. *Shock. Vib.* **2021**, 7937459. 10.1155/2021/7937459 (2021).

[47] Ran, Q. C. et al. Experimental investigation on mechanical characteristics of red sandstone under graded cyclic loading and its inspirations for stability of overlying strata. *Geomech. Geophys. Geo-Energy Geo-Resour.* **9**, 11. 10.1007/s40948-023-00555-x (2023).

[48] Zhang, B. C., Liang, Y. P., Zou, Q. L., Ding, L. Q. & Ran, Q. C. Experimental investigation into the damage evolution of sandstone under decreasing-amplitude stress rates and its implications for coalbed methane exploitation. *Environ. Earth Sci.* **82**, 208. 10.1007/s12665-023-10825-2 (2023).

[49] Zhang, B. C. et al. Effect of stress amplitude on mechanical and acoustic emission of sandstone under constant–cyclic loading. *Bull. Eng. Geol. Env.* **82**, 284. 10.1007/s10064-023-03307-z (2023).

[50] Wang, Z. L. et al. Evolution mechanism and quantitative characterization of initial micro-cracks in marble under triaxial compression. *J. Zhejiang Univ. –Sci. A* **25**, 586–595. 10.1631/jzus.A2300159 (2024).

[51] Ran, Q. C. et al. Failure mechanisms of sandstone subjected to cyclic loading considering stress amplitude effects. *Int. J. Coal Sci. Technol.* **12**, 68. 10.1007/s40789-025-00802-4 (2025).

[52] Xu, Y., Li, C. J., Zheng, Q. Q., Ni, X. & Wang, Q. Q. Analysis of energy evolution and damage characteristics of mudstone under cyclic loading and unloading. *Chin. J. Rock Mech. Eng.* **38**, 2084–2091. 10.13722/j.cnki.jrme.2019.0153 (2019).

[53] Yin, D. W. et al. Experimental study on mechanical properties of coal soaked in pressurized water considering initial damage. *J. China Coal Soc.* **48**, 4417–4432. 10.13225/j.cnki.jccs.2023.0256 (2023).

[54] Wang, Z. L., Li, S. Y., Wang, J. G., Xiong, F. & Xie, L. X. Mechanical behavior, mesoscopic properties and energy evolution of deeply buried marble during triaxial loading. *Int. J. Damage Mech* **31**, 1592–1612. 10.1177/10567895221107707 (2022).

[55] Du, X. H. et al. Triaxial mechanical behaviour and energy conversion characteristics of deep coal bodies under confining pressure. *Energy* **266**, 126443. 10.1016/j.energy.2022.126443 (2023).

[56] Li, S. G. et al. Relationship between micro-pores fractal characteristics about NMR T2 spectra and macro cracks fractal laws based on box dimension method of coal under impact load from energy dissipation theory. *Chaos, Solitons Fractals* **189**, 115685. 10.1016/j.chaos.2024.115685 (2024).

[57] Li, S. Y. et al. Failure characteristics and brittleness index establishment based on marble energy evolution mechanism. *Geomech Energy Envir* **36**, 100504. 10.1016/j.gete.2023.100504 (2023).

[58] He, D. et al. Effects of dynamic impact on microstructure evolution and permeability enhancement of coal. *Energy Fuels* **39**, 11099–11109. 10.1021/acs.energyfuels.5c01419 (2025).

[59] Ma, T. F. et al. Regulation mechanism of surfactant-SiO_2_ nanoparticle compound on the mechanical property and microstructure of coal: Effect of the type of surfactant. *Powder Technol.* **466**, 121459. 10.1016/j.powtec.2025.121459 (2025).

[60] Li, S. G. et al. Deformation and seepage characteristics of gassy coal subjected to cyclic loading-unloading of pore pressure. *Nat. Resour. Res.* **34**, 2775–2796. 10.1007/s11053-025-10541-7 (2025).

[61] Wang, C. Z. et al. Dominant governing mechanisms of deformation-seepage and dynamic evolution model of permeability in gas-containing coal under coupled stress-pore pressure. *Fuel* **404**, 136408. 10.1016/j.fuel.2025.136408 (2025).

[62] Zou, Q. L., Ma, T. F., Liang, J. Y., Xu, B. C. & Ran, Q. C. Mesomechanical weakening mechanism of coal modified by nanofluids with disparately sized SiO2 nanoparticles. *Int. J. Rock Mech. Min. Sci.* **188**, 106056. 10.1016/j.ijrmms.2025.106056 (2025).

[63] Ran, Q. C., Zhao, W. T., Liang, Y. P., Ye, C. F. & Ning, Y. H. Deciphering confining pressure effects on coal failure mechanisms using acoustic emission approaches. *Eng. Fract. Mech.* **328**, 111592. 10.1016/j.engfracmech.2025.111592 (2025).

[64] Shan, P. F. et al. Study on acoustic emission rate response of coal samples considering initial damage difference. *J. Min. Safe. Eng.* **40**, 798–808. 10.13545/j.cnki.jmse.2022.0295 (2023).

[65] Chang, X. K., Wu, S. C., Zhang, Z. R. & Dai, F. Investigating the simulation test and acoustic emission characteristics of structural-control type rockbursts in deep underground environments. *Eng. Fract. Mech.* **310**, 110477. 10.1016/j.engfracmech.2024.110477 (2024).

[66] Yang, H. Z. et al. Predicting the failure of rock using critical slowing down theory on acoustic emission characteristics. *Eng. Fail. Anal.* **163**, 108474. 10.1016/j.engfailanal.2024.108474 (2024).

[67] Lu, J., Jiang, W., Xie, H. P., Gao, H. & Zhang, D. M. Dynamic disaster mechanism and acoustic emission evolution of deep coal-rock under true triaxial disturbance stress. *J. Rock Mech. Geotech. Eng.*10.1016/j.jrmge.2024.12.014 (2025).

[68] Ye, C. F. et al. Progressive failure mechanism and shear strength model of granite under cyclic direct shear. *Rock Mech. Rock Eng.*10.1007/s00603-025-04962-2 (2025).

[69] Ran, Q. C. et al. Statistical fractal pattern of acoustic emission and failure precursor characterization in damaged coal subjected to cyclic loading. *J. China Coal Soc.* **50**, 2728-2736, 10.13225/j.cnki.jccs.2025.0578 (2025).

[70] Zhou, Z. L., Zhao, T. H., Ullah, B. & Fan, J. L. Investigating crack evolution, and failure precursor warning in sandstones with different water contents from the perspective of tensile-shear crack separation. *Eng. Fail. Anal.* **167**, 108997. 10.1016/j.engfailanal.2024.108997 (2025).

[71] Yu, X. et al. Uncovering the progressive failure process of primary coal-rock mass specimens: Insights from energy evolution, acoustic emission crack patterns, and visual characterization. *Int. J. Rock Mech. Min. Sci.* **178**, 105773. 10.1016/j.ijrmms.2024.105773 (2024).

[72] He, Q. C. et al. Compressive failure patterns and acoustic emission characteristics of reservoir rocks subjected to chemical corrosion for underground energy storage. *J. Energy Storage* **170**, 114950. 10.1016/j.est.2024.114950 (2025).

[73] Gao, R. B. et al. Influence of coal-to-concrete height ratio on progressive damage characteristics of composite structures: Acoustic emission analysis. *Phys. Fluids* **37**, 057127. 10.1063/5.0272252 (2025).

[74] Ran, Q. C. et al. Hardening-damage evolutionary mechanism of sandstone under multi-level cyclic loading. *Eng. Fract. Mech.* **307**, 110291. 10.1016/j.engfracmech.2024.110291 (2024).

[75] Xie, Y. C., Hou, M. Z. & Li, C. B. Anisotropic characteristics of acoustic emission and the corresponding multifractal spectrum during progressive failure of shale under cyclic loading. *Int. J. Rock Mech. Min. Sci.* **165**, 105364. 10.1016/j.ijrmms.2023.105364 (2023).

[76] Zhang, K. et al. Experimental study on acoustic emission evolution characteristics and response mechanism of damaged rocks. *Coal Geol. Explor.* **52**, 96–106. 10.12363/issn.1001-1986.23.09.0548 (2024).

[77] Ran, Q. C. et al. Failure analysis of overlying strata during inclined coal seam mining: Insights from acoustic emission monitoring. *Eng. Fail. Anal.* **182**, 110023. 10.1016/j.engfailanal.2025.110023 (2025).

